# Identification of the Potential Prognostic Markers from the miRNA-lncRNA-mRNA Interactions for Metastatic Renal Cancer via Next-Generation Sequencing and Bioinformatics

**DOI:** 10.3390/diagnostics10040228

**Published:** 2020-04-16

**Authors:** I-Jeng Yeh, Kuan-Ting Liu, Jheng-Heng Shen, Yen-Hung Wu, Yao-Hua Liu, Meng-Chi Yen, Po-Lin Kuo

**Affiliations:** 1 Graduate Institute of Clinical Medicine, College of Medicine, Kaohsiung Medical University, Kaohsiung 807, Taiwan; ijengyeh@hotmail.com (I.-J.Y.); kuantingliu7@gmail.com (K.-T.L.); blan32705@hotmail.com (Y.-H.W.); 2 Department of Emergency Medicine, Kaohsiung Medical University Hospital, Kaohsiung Medical University, Kaohsiung 807, Taiwan; Youngfoot_1017@yahoo.com.tw (J.-H.S.); lau0619@yahoo.com.tw (Y.-H.L.); 3 School of Medicine, College of Medicine, Kaohsiung Medical University, Kaohsiung 80708, Taiwan; 4 Ph.D. Program in Environmental and Occupational Medicine, College of Medicine, Kaohsiung Medical University, Kaohsiung 80708, Taiwan; 5 Institute of Medical Science and Technology, National Sun Yat-Sen University, Kaohsiung 80708, Taiwan

**Keywords:** kidney cancer, renal cancer, metastasis, RNA sequencing, small RNA sequencing, long non-coding RNA (lncRNA), microRNA (miRNA)

## Abstract

The survival rate in patients with metastatic renal cell carcinoma (RCC) is low. In addition, metastatic RCC resists traditional treatment. Therefore, identification of novel biomarkers, signaling pathways, and therapeutic targets is an important issue. The aim of the present study is to identify novel prognostic markers from the miRNA-mediated network for the regulation of metastasis of RCC. To address this issue, the RNA of human RCC cell lines, 786-O and ACHN, derived from primary and metastatic sites, respectively, were collected and subjected to RNA sequencing and small RNA sequencing. The bioinformatic analysis revealed that the pathways of the genes with different expressions were related to tumor progression, and identified miRNA and miRNA-long non-coding RNA (lncRNA) interactions, and mRNA. The results revealed that the expressions of seven miRNAs were associated with the overall survival rate of patients with RCC. Furthermore, the expressions of two lncRNA and three protein-coding genes (mRNA) were significantly associated with the increased or decreased disease-free survival rate. Although the detailed regulatory mechanism between miRNAs and targeted genes was not fully understood, our findings present novel prognostic markers and novel insight on miRNA-mediated pathways for metastatic RCC.

## 1. Introduction

Renal cell carcinoma (RCC) is one of the 10 most common types of cancer in the world [1]. It can be classified into different subtypes, such as clear cell RCC (approximately 70–80% patients with RCC), papillary RCC, and chromophobe RCC, among others [2]. Surgical resection is the preferred treatment for localized RCC. However, 20–30% of metastases occur several years after surgery [3]. Clinical data revealed that the five-year disease-specific survival for clear cell RCC and papillary RCC is 10.5% and 10.3%, respectively once metastasis occurs [4]. Metastatic RCC resists chemotherapy and radiotherapy treatment. Therefore, development of novel treatments is an important issue.

Hypoxic signaling is a driving force in tumor progression and early stage metastasis [5]. Hypoxia-inducible factor (HIF)-1 signaling pathway is activated under hypoxic stress and leads to enhanced tumor growth, angiogenesis, and metastasis in several types of cancers, including RCC [6,7]. Over 100 HIF-responsive genes, such as vascular endothelial growth factor (VEGF) and platelet-derived growth factor (PDGF) are identified [8]. In addition, downstream of the VEGF receptor (VEGFR) pathway, the mammalian target of rapamycin (mTOR) kinase, is demonstrated as a critical mediator of hypoxic responses [9,10]. Based on these findings, administration of targeted therapy agents have been used in the first line of treatment of recurrent RCC [11,12]. Tyrosine kinase inhibitors, such as sunitinib, pazopanib, and bevacizumab, are approved for metastatic RCC treatment [12,13]. The mTOR inhibitors, temsirolimus and everolimus, are authorized for treatment of metastatic RCC [14]. Furthermore, there are multiple types of monotherapy and combination therapy with targeted drugs in preclinical and clinical trials. Thus, identification of novel molecules involved in metastasis of RCC is beneficial for the development of a novel targeted drug.

MicroRNAs (miRNAs) are a kind of small non-coding RNAs involving in many biological processes, including tumorigenesis and metastasis. Thus, miRNAs regulating tumor metastasis may be attractive therapeutic targets [15]. In RCC, miRNA may serve as biomarkers for identifying the signatures of poor prognosis and poor therapeutic responses in patients [16]. The recent study reveals that miRNAs and miRNA-targeted messenger RNA (mRNA) are deeply involved in tumor progression and metastasis in RCC [17]. Moreover, some specific long non-coding RNA (lncRNA, known as long intergenic noncoding RNA) genes are demonstrated to serve as biomarkers for diagnosis and prognosis, to serve as therapeutic targets, and to regulate tumorigenesis and metastasis of RCC [18,19]. Therefore, comprehensive analysis of miRNA, lncRNA, and mRNA networks would be beneficial for clinical application. Here, the expression patterns were systematically detected in the RCC line isolated from primary tumor and metastatic site via small RNA sequencing and RNA sequencing. The miRNA-interacting protein-coding gene and long non-coding RNA genes, and the potential prognostic markers in metastatic RCC were determined.

## 2. Materials and Methods

### 2.1. Cell Culture

786-O (ATCC^®^ CRL-1932) and ACHN (ATCC^®^ CRL-1611™) are human RCC cell lines derived from a primary renal tumor and a metastatic site, respectively. These cell lines were obtained from American Type Culture Collection (Manassas, VA, USA). Next, 786-O and ACHN were cultured in RPMI-1640 medium and Eagle’s Minimum Essential Medium, respectively, which were supplied with 10% fetal bovine serum (FBS) and 10,000 units penicillin, 10 μg streptomycin, and 25 μg amphotericin B per ml (Gibco; Thermo Fisher Scientific, Inc., Waltham, CA, USA). The cells were incubated at 37 °C and 5% CO_2_ atmosphere.

### 2.2. Preparation of RNA Sequencing

2 × 10^6^ 786-O and ACHN cells were seeded into a 10 cm dish. After 24 h, the total RNA was collected and then extracted using TRIzol^®^ reagent (Thermo Fisher Scientific, Inc.). Each group contained one RNA sample. RNA concentration and quality were determined via an ND-1000 spectrophotometer (NanoDrop Technologies; Thermo Fisher and an Agilent 2100 Bioanalyzer and an RNA 6000 Pico LabChip RNA (Agilent Technologies, Inc, Santa Clara, CA, US.), respectively. The quality report is shown in Figure S1. The RNA sample was subjected to RNA sequencing and small RNA sequencing.

### 2.3. Sequencing, Alignment, and Differential Gene and miRNA Expressions

The library construction, RNA sequencing, and small RNA sequencing were accomplished by Welgene Biotech Co., Ltd. (Taipei, Taiwan). Sequencing quality trimming, mRNA alignment, and miRNA alignment were performed via Trimmomatic version 0.36 [20], HISAT2 [21], and miRDeep2 [22]. 

### 2.4. miRNA Enrichment Analysis

The criteria of significantly differential miRNA expressions were set at fold change ≥5.0 and reads per million (RPM) >1. To determine the enriched pathways, the two bioinformatic tools used were the GSEA method of miRNA Enrichment Analysis and Annotation Tool (miEAA; https://ccb-compute2.cs.uni-saarland.de/mieaa_tool/) [23] (the parameter of analysis was set at “Pathways (miRWalk)”, *p* value adjustment method: FDR adjustment, Significance level: 2, and Threshold level: 2), and Funrich software version 3.1.3 (miRNA enrichment, biological pathway) [24].

### 2.5. Gene Ontology (GO) Analysis of Protein-Coding Genes

To determine the biological function of protein-coding genes enriched in ACHN cells, the biological process of GO (GOTERM_BP_FAT) analysis was performed via DAVID Bioinformatics Resources 6.8 (https://david.ncifcrf.gov/home.jsp) [25]. The threshold was set as count: 2 and EASE score: 0.1.

### 2.6. Prediction of miRNA-Targeted Genes

The miRNA-targeted genes were predicted by miRNet 2.0 (https://www.mirnet.ca/miRNet/home.xhtml) [26] and Funrich software 3.1.3. To search for the targets of miRNAs, the parameter was set as organism “*H. sapiens* (human)”, ID type “miRBase ID”, Tissue (human only) “Not specified”, and Targets “Genes” and ”lncRNAs”. In addition, information on the experimental validation for interaction of miRNA and its targeted gene was also obtained from miRNet 2.0. The miRNA-gene interactions of miRNet 2.0 database [26] was collected from The miRNA target gene data were collected from well-annotated database miRTarBase v7.0 [27], TarBase v7.0 [28], and miRecords [29]. In addition, the information on experimental validation was also collected from miRTarBase v7.0. The miRNet-genes interaction from Funrich software was based on the Funrich database itself. These interacting genes that were found in both databases were considered as miRNA-interacting genes.

### 2.7. Selection of Genes with Differential Expression

The criteria of significantly differential protein-coding gene (mRNA) expressions were set at fold change ≥10.0 and reads per kilobase million (RPKM) >0.0001. 

### 2.8. Assessment of Gene Expression and Patient’s Prognosis

The association between expression of each microRNA and overall survival rate of three types of RCC was evaluated by OncomiR (http://www.oncomir.org) [30]. The expressions of each gene in samples of kidney renal clear cell carcinoma, kidney renal papillary cell carcinoma, and kidney chromophobe in the disease-free survival analysis were evaluated by GEPIA2 database (http://gepia2.cancer-pku.cn/#index) [31].

### 2.9. Determine the Gene Expression from Online Datasets

The expression of each gene in metastatic tumor samples and primary tumor samples was evaluated by HCMDB (http://hcmdb.i-sanger.com/index) [32]. The expression values were obtained from two datasets (GEO accession number: GSE22541 and GSE85258).

### 2.10. Statistical Analysis

The *p* value adjustment of miRNA enrichment analysis ((G)SEA)) on the miEAA website was based on the FDR adjustment method. *p* value <0.05 and threshold >2 was considered significant. The *p* value of Funrich software was shown by Bonferroni correction. The logrank *p* value of OncomiR results from a univariate Cox analysis. The logrank test (Mantel-Cox method) was used for survival analysis on the GEPIA2 database. *p* value <0.05 was considered statistically significant.

## 3. Results

### 3.1. The Results of miRNA Sequencing

**Figure 1:**
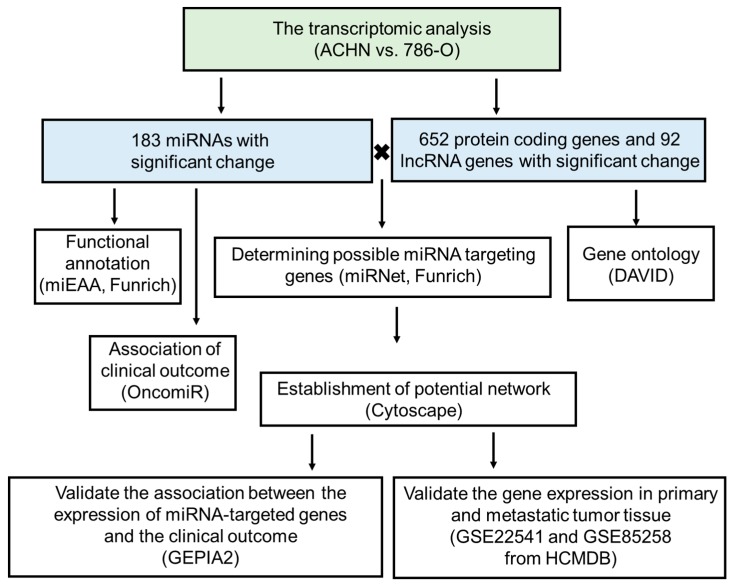
Flowchart of this study. The RNA of 786-O and ACHN cells were extracted and then subjected to RNA and small RNA sequencing. The differentially expressed 183 miRNA genes, 652 protein coding genes, and 92 lncRNA genes were identified. The functional annotation were performed via miRNet, Funrich, and DAVID. The possible miRNA targeting genes were predicted by miRNet and Funrich. The association of gene expression and survival rate was evaluated by OncomiR and GEPIA2. The expression in primary and metastatic tumor tissue via GEO datasets (the fold change and statistical analysis of GSE22541 and GSE85258 was adapted from HCMDB).

To investigate the difference of regulatory networks between the primary and metastatic tumor, two human RCC cell lines, 786-O, and ACHN derived from primary and metastatic (derived from pleural effusion) sites respectively, were chosen in this study. Total RNA of each cell line (one sample in each group) was extracted from both cells 24 h after seeding and was subjected to RNA sequencing and small RNA sequencing. The result of extracted RNA quantity assessment was shown in Figure S1. The high score in per-base sequence quality and per-sequence quality was observed in each group. To further investigate the differential miRNA expressions in primary and metastatic RCC cell lines, miRNAs with fold change ≥5.0 or ≤−5.0 and RPM >1 were considered significant. Based on these criteria, 183 mature miRNA were selected for the following analyses (the complete miRNA list and its fold changes are presented in Table S1). Besides, the results of RNA sequencing identified 652 protein-coding genes and 92 lncRNA genes with significantly different gene expressions (fold change ≥10 or ≤−10.0, FPKM >0.0001) between ACHN and 786-O. The list of mRNA and lncRNA and the fold changes were presented in Tables S2 and S3, respectively. The results were further analyzed by bioinformatic methods. The flowchart of this study was shown in Figure 1. The detailed settings of the bioinformatic databases were listed in the method sections.

### 3.2. The miRNA Enrichment Analysis of Differential miRNA Expressions

**Figure 2:**
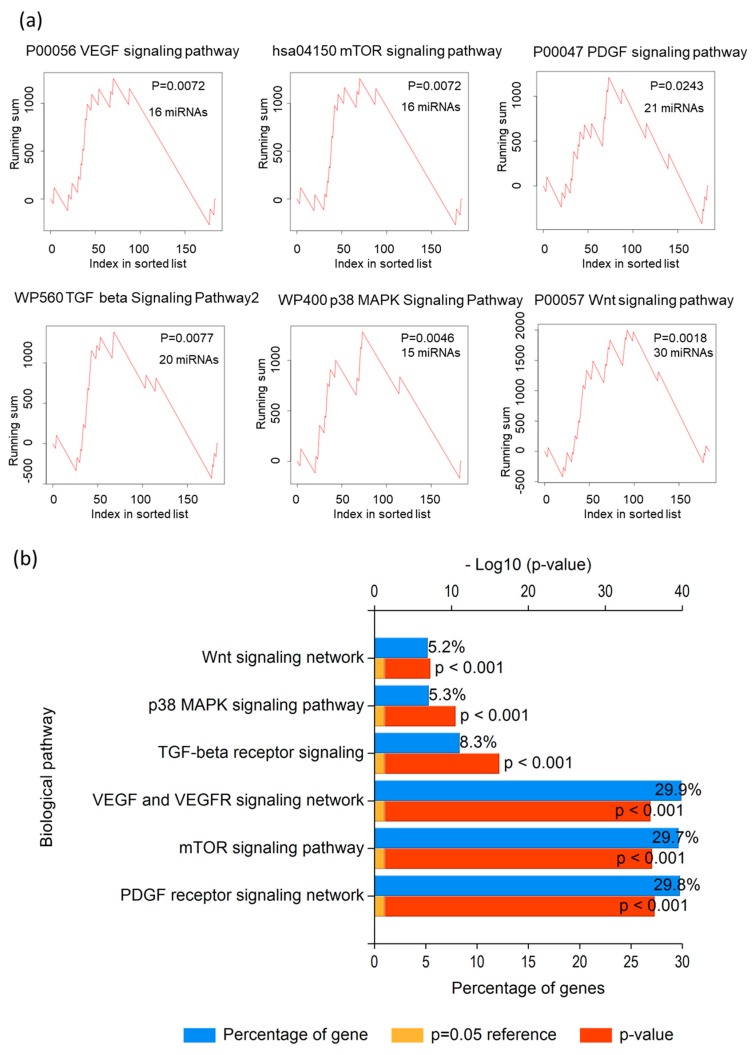
Identified statistically significantly enriched pathways, via (**a**) miEAA website (miRNA enrichment analysis, pathways (miRWalk); and (**b**) Funrich software (biological pathways).

Previous studies indicated that the VEGF, PDGF, mTOR signaling pathways could serve as therapeutic targets for metastatic RCC in clinical practice [8,9,10]. To investigate whether the miRNAs with 183 significantly different miRNA expressions were involved in these pathways, the miRNA enrichment analysis was further determined via two bioinformatic tools, miEAA website (miRNA enrichment analysis ((G)SEA)), and Funrich software (biological pathways). More than 100 biological pathways were identified as statistically significant enriched pathways (*p* value < 0.05), including EGF, PDGF, and mTOR signaling pathways. In addition, some similar signaling pathways were identified through bioinformatic analysis, including the Wnt signaling pathway, ErbB signaling pathway, integrin signaling pathway, p38 MAPK signaling pathway, the RCC, and TGF beta signaling pathway. The recent studies have shown that Wnt/β-catenin pathway [33], the MAPK signaling pathway, and ErbB signaling pathway [34] are associated with kidney cancers; TGF beta signaling pathway and integrin signaling pathway are involved in regulating epithelial to mesenchymal transition (EMT) in RCC [35]. The list of nine signaling pathways and miRNAs was shown in Table 1 and Figure 2. The list of the other enriched pathways was listed in Table S4 (*p* value < 0.03 was shown).

### 3.3. The Gene Ontology (GO) Analysis of Differential Protein-Coding Gene Expressions

GO analyses of 652 protein-coding genes were performed using DAVID. When compared to 786-O, the top ten significant enriched biological processes in ACHN were cell adhesion, biological adhesion, extracellular matrix organization, extracellular structure organization, growth, cell migration, developmental growth, cell morphogenesis involved in differentiation, regulation of cell motility, cell motility, and localization of cell (Table 2). The significant biological process was shown in Table S5 (*p* value < 0.0001).

### 3.4. Identification of Potential miRNA-Targeting mRNA and lncRNA

**Figure 3:**
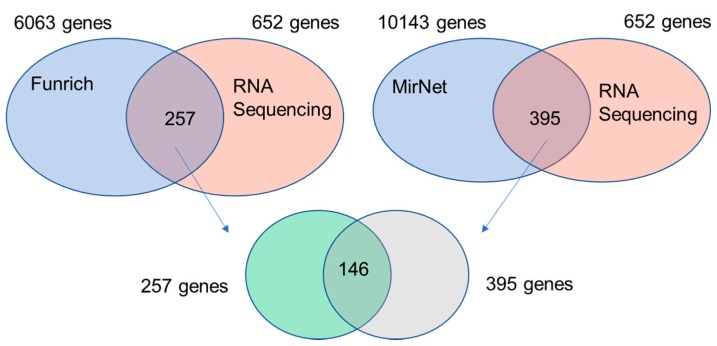
Venn diagram analysis indicating the identification of shared miRNAs-targeted protein-coding genes. Blue arrows indicate the 257 genes and 395 genes in the lower panel were from the intersected genes between Funrich and RNA sequencing, and miRNet and RNA sequencing respectively.

To further identify the potential targeted genes of 183 miRNAs, the Funrich software and miRNet (an integrated platform linking miRNA, targets, and functions) were used. A total of 6,063 and 10,143 of 183 miRNAs-targeted protein-coding genes were predicted via Funrich software and miRNet, respectively. Besides, the results of RNA sequencing revealed 652 protein-coding genes and 92 lncRNA genes with significant differences between ACHN and 786-O. The intersection between 652 protein-coding genes and predicted 183 miRNA-targeted genes revealed 257 genes by Funrich software and 395 genes by miRNet, respectively. Another 146 shared genes were subsequently identified (Figure 3). In addition, the intersection between 92 lncRNAs and miRNet-predicted targeted lincRNAs included Long Intergenic Non-Protein Coding RNA 472 (LINC00472), Nuclear Paraspeckle Assembly Transcript 1 (NEAT1), and Colon Cancer Associated Transcript 1 (CCAT1).

### 3.5. Assessment of whether the miRNA Expression Was Associated with the Survival Outcome in Patients with RCC

To assess whether the miRNAs that regulate 146 protein genes and 3 lncRNA genes serve as a prognostic marker, the OncomiR, an online resource for exploring pan-cancer microRNA dysregulation, was used for screening the association between miRNA expression and clinical outcome of patients with RCC. Increased expressions of the seven following miRNAs were detected in ACHN: hsa-miR-137, hsa-miR-224-5p, hsa-mir-296-3p, hsa-mir-335-5p, hsa-miR-34c-5p, hsa-mir-377-3p, and hsa-miR-153-3p. In addition, the seven miRNAs were up-regulated in deceased patients with different types of RCC, including kidney chromophobe, kidney renal clear cell carcinoma, and kidney renal papillary cell carcinoma (Table 3).

### 3.6. Evaluation of the Potential Interaction of miRNA and Genes

The expression patterns of seven miRNA and their targeted genes (precited by miRNet), and the potential interaction are listed in Table 4. Moreover, the experimental validation and literature of each interaction are listed in Table 4.

### 3.7. Validation of the Association of Predicted miRNA-Targeted Gene Expression and Survival Outcome

**Figure 4:**
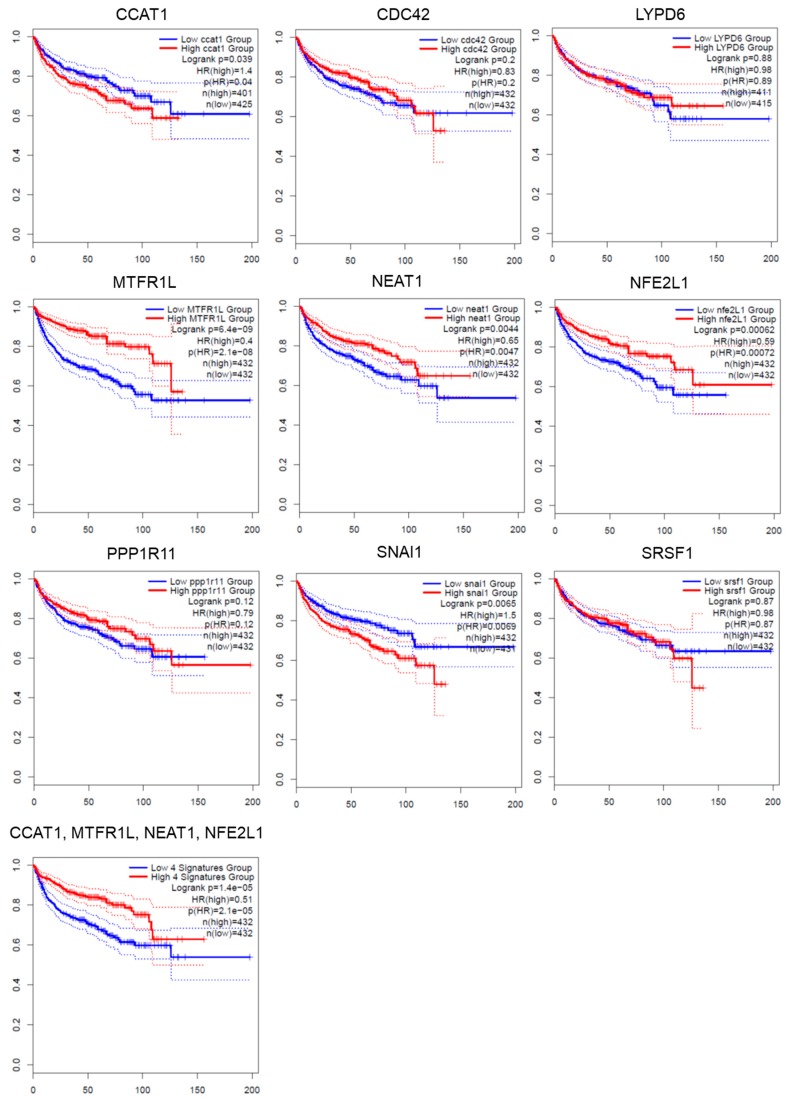
The disease-free survival comparing the high expression (red line) and low expression (blue line) of patients with kidney renal clear cell carcinoma, kidney renal papillary cell carcinoma, and kidney chromophobe were created from data in the GEPIA2 database. The gene expression was divided by median expression value. The number in parentheses indicated the number of groups. The logrank *p* value and hazard ratio (HR) was shown on each figure. 95% confidence interval was shown as dotted line.

**Figure 5:**
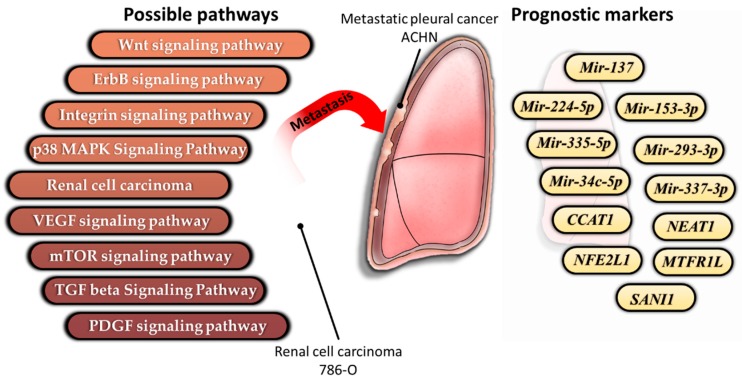
Schematic summary of the identified molecules in metastatic RCC cells.

To further validate whether the miRNA-targeted genes were associated with relapse-free survival in patients with kidney cancers, it was evaluated via the GEPIA2 database (Figure 4). Nine genes, including long non-coding RNA *Nuclear Paraspeckle Assembly Transcript 1 (NEAT1)* and *Colon Cancer Associated Transcript 1 (CCAT1)*, protein coding genes *Cell Division Cycle 42 (CDC42), Protein Phosphatase 1 Regulatory Inhibitor Subunit 11 (PPP1R11), Nuclear Factor, Erythroid 2 Like 1 (NFE2L1), Mitochondrial Fission Regulator 1 Like (MTFR1L), LY6/PLAUR Domain Containing 6 (LYPD6)*, *Serine And Arginine Rich Splicing Factor 1 (SRSF1)*, and *Snail Family Transcriptional Repressor 1 (SNAI1)* were evaluated. In Figure 5, the trend toward improved disease-free survival was observed in patients with kidney cancer with a higher expression of *CCAT1*, *NEAT1, MTFRL1,* and *NFE2L1.* The opposite pattern of disease-free survival was shown in *SNAI1*. The Kaplan–Meier curve was also performed according to the expression of a four gene signature.

To further evaluate whether the expression levels of *NEAT1*, *MTFRL1*, *NFE2L1*, and *SANI1* in metastatic tumor samples were different from those in primary tumor samples, the expression of the above genes in metastasis tumor tissues and primary tumor tissues were examined via two datasets of Gene Expression Omnibus (GEO) (Table 5). The dataset GSE22541 contained 43 pulmonary metastatic tumor tissues and 17 primary tumor tissues from the lungs of patients with clear cell RCC, and the dataset GSE85258 contained 15 pairs of primary RCC tumors (14 kidney renal clear cell carcinoma and 1 kidney renal papillary cell carcinoma) and matched pulmonary metastases. The results showed that the expression of *NFE2L1* in metastatic tumor was lower than that in the primary tumor (*p* value < 0.05) in the GSE22541 dataset. Relatively low expression of *MTFR1L* was observed in GSE85258 dataset. The expression patterns of *NFE2L1*, and *MTFR1L* in both datasets were consistent with those in the results of RNA sequencing. Besides, the data of CCAT1 expression can’t be found in either GSE22541 or GSE85258 datasets. The summary of this study was shown in Figure 5.

## 4. Discussion

The aim of the present study was to identify novel prognostic biomarkers from the interacting genes of miRNA in metastatic RCC. From the results of small RNA and RNA sequencing in a primary kidney cancer cell line 786-O, 183 miRNAs, 652 protein-coding genes, and 92 lncRNA genes were identified. The intersection of the 183 predicted miRNAs-targeted genes and RNA sequencing-identified protein coding and lncRNA genes revealed 146 protein-coding genes and 3 lncRNAs. After matching the expression patterns of miRNAs and miRNA-targeted genes, the potential interacting genes of miRNA were listed in Table 3.

The miRNA-enrichment analysis showed that 183 miRNAs were involved in various physiological pathways. In addition, some known pathways, such as VEGF, PDGF, and mTOR signaling pathways, are demonstrated to be therapeutic targets for metastatic RCC [8,9,10]. The other pathways, including the Wnt/β-catenin pathway, MAPK signaling pathway, ErbB signaling pathway, TGF-beta signaling pathway, and integrin signaling pathway also play important roles in the progression of kidney cancer [33,34,35]. In addition, the GO of protein-coding genes revealed that enriched biological processes, such as adhesion, growth, and cell motility, were associated with metastasis of RCC. This may imply that the enriched pathways of these identified miRNAs and protein-coding genes (mRNAs) from RCC lines are similar to the enriched-physiological and pathological pathways in human renal carcinoma.

To identify the possible prognostic markers in interacting genes of miRNAs, seven miRNAs (hsa-miR-137, hsa-miR-224-5p, hsa-mir-296-3p, hsa-mir-335-5p, hsa-miR-34c-5p, hsa-mir-377-3p, and hsa-miR-153-3p) were identified because the high expression of these genes were associated with a poor overall survival rate in different types of RCC (Table 3). The seven miRNAs were also involved in at least one of nine signaling pathways listed in Table 1. Therefore, we supposed that the seven miRNAs may serve as biomarkers for prediction of patients’ clinical outcomes.

Before the present study, various studies demonstrated that miRNAs such as hsa-miR-137 [54,55], hsa-mir-211-5p [56], hsa-mir-224-5p [57], hsa-miR-335-5p [58], and hsa-mir-296-3p [59], are involved in the regulation of RCC progression. In addition, increased hsa-miRNA-9 and decreased hsa-miRNA-200a could serve as biomarkers for distinguishing hemangioblastomas from metastatic clear cell RCC [60]. The molecular function of some miRNAs in RCC has been determined in previous studies. The current studies suggest that has-miR-137 may serve as a suppressor of tumor progression and metastasis in RCC [54,55]. Besides, the tumor suppressor function of hsa-miR-224-5p and hsa-miR-335-5p is also demonstrated in clear cell RCC [57,58]. In contrast, hsa-miR-296-3p was demonstrated in migration and invasion in clear cell RCC [59]. The roles of hsa-miR-34c-5p, hsa-miR-377-3p, and hsa-miR-153-3p in RCC were not fully determined by experimental evidence. It is interesting to note that the experimental evidence suggests tumor-suppressive roles for has-miR-137, hsa-miR-224-5p, and hsa-miR-335-5p. However, high expression of the above miRNAs are associated with poor prognosis in different types of RCC according to the RNA sequencing data of clinical RCC samples and our sequencing data. We supposed that the expression level of miRNAs may only serve as a potential biomarker for RCC progression but may not be associated with the molecular function for tumor progression. It is worth further investigating the miRNA-mediated functions in clinical samples, especially metastatic RCC samples.

Apart from miRNAs, our results identified that the expression of two lncRNA (*NEAT1* and *CCAT1*) and three protein-coding genes (*NFE2L1*, *MTFR1L*, and *SNAI1)* were significantly associated with increased or decreased disease-free survival in patients with RCC. In addition, the expression of *NFE2L1* and *MTFR1L* in metastatic tumor samples is lower than that in primary tumor samples. LncRNA *NEAT1* knock-down by siRNA results in suppression of proliferation and epithelial-mesenchymal transition-related markers in clear cell RCC cell lines [61]. In addition, the oncogenic role of CCAT1 is reported in multiple types of human cancer [62]. Silencing CCAT1 expression represses the viability of RCC [63]. Therefore, high expression of *NEAT1* and *CCAT1* is associated with oncogenic phenotypes in RCC. However, the molecular functions of *CCAT1* and *NEAT1* are not identical with those in the results of disease-free survival. This needs to be addressed in future investigations. The *SNAI1* gene encodes transcription factor SNAIL, which promotes metastatic phenotypes in RCC. Inhibition of *SNAI1* via miR-211-5p suppresses metastatic behavior in RCC [56]. The TOX High Mobility Group Box Family Member 3 (TOX3) protein is a repressor for inhibition of SNAIL proteins encoded by *SNAI1* and *SNAI2* in clear cell RCC [64]. Therefore, molecular SNAI1 tend to promote cell migration and invasion. High *SNAI1* expression is associated with a poor survival rate. The NFE2L1 (also referred to as NRF1) protein has been implicated in cancer development, and degenerative, and metabolic disorders [65]. However, the molecular functions of *NFE2L1* and *MTFR1L* have not been investigated in RCC. It is worth addressing the detailed molecular function and regulatory mechanism of RCC in vitro and in vivo.

Besides NFE2L1 and MTFR1L, the expression patterns of three other genes in two GEO datasets were not identical with our results. The most metastatic sites for RCC are the lungs, lymph nodes, liver, brain, and bone [66,67]. The metastatic tumor samples in both GEO datasets were collected from the lung. Because ACHN is derived from pleural effusion, the gene expression pattern of ACHN may be different from that of other metastatic sites. In addition, the gene expression pattern in a cell line could not represent the gene expression pattern in clinical samples. This may be a possible reason for inconsistent gene expression between our sequencing data and metastatic tissues of RCC.

Hypoxic stress is a critical factor in tumor progression. Hypoxia Inducible Factor 1 Subunit Alpha (*HIF1A*) gene encodes transcription factor hypoxia-inducible factor-1, which is a master regulator for homeostatic response to hypoxia. Regulation of HIF1A is important for RCC development [68]. In our previous study, the decreased expressions of miRNA hsa-mir-100 and hsa-mir-378 were observed in 786-O under long-term hypoxic culture [69]. In addition, the migration ability of 786-O was enhanced after long-term hypoxic culture [69]. In the present study, the predicted interacting genes did not show interaction between HIF1A, hsa-mir-100, and hsa-mir-378. Therefore, the genes enriched in ACHN were different from those in long-term hypoxia-treated 786-O.

Investigation of the miRNA-genes and miRNA-lncRNAs interactions may be applied on various research fields in the future. Since miRNAs are involved in cancer-associated processes, they can serve as monotherapy or adjuvant therapy for cancer treatment and management [70]. For example, Lai et al. demonstrated that enhanced expression of miR-205-5p and miR-342-3p results in decreased tumor chemoresistance by cooperatively repressing E2F1 [71]. In the present study, high expressions of the seven identified miRNAs were enriched in patients died of RCC. Repressing the expression of seven miRNAs may be a strategy for RCC treatment and the effect of repression of miRNAs can be predicted via miRNA-interacting genes in metastatic RCC.

The information from RNA sequencing is a novel tool for improving clinical diagnostics [72]. Thus, the present study tried to find novel biomarkers and potential therapeutic markers for metastatic RCC. However, the limitations of the present study should be noted. First, the predicted miRNA-interacting genes were constructed via sequencing data from RCC cell lines. Although the gene ontology analysis of miRNAs (Table 1 and Figure 2) and mRNAs (data not shown) showed various enriched pathways involved in progression and metastasis of human RCC, the regulatory mechanism needs to be determined by experimental evidence in vitro, in animal tumor models, or clinical RCC samples in subsequent studies. Second, gene ontology analysis of miRNA, protein-coding genes, and lncRNAs was performed according to the criteria for gene selection (such as fold change and read counts). The results of ontology analysis may be affected by this subjective methodology. In addition, some important biomarkers may be ignored by our analytical methods. The third limitation is that the miRNA-mediated pathways were also affected by epigenetic regulation. Because RNA and small RNA sequencing could not detect epigenetic changes, the effect of epigenetic regulation on these pathways and metastasis of RCC could not be considered in the study. The fourth limitation is that the miRNA-interacting genes were predicted by gene expression. Integration of mathematical modelling and bioinformatic methods is essential for improving an understanding of the function of miRNA and miRNA-mediated network [73].

## 5. Conclusions

It is not easy to obtain the clinical tumor samples from metastatic RCC. Alternatively, the present study tries to identify novel miRNA-interacting genes through RNA-seq of two single RCC cell lines. The expression patterns of these identified genes were further validated in clinical RCC samples on multiple public databases. The different expression lncRNA (*NEAT1* and *CCAT1*) and three protein-coding genes (*NFE2L1*, *MTFR1L*, and *SNAI1*) have been identified, but the molecular function was not well-known in RCC. With the exception of identifying potential prognostic markers, our study may be beneficial in the development of novel therapeutic strategies for treatment of metastatic RCC in the future.

## Figures and Tables

**Table 1:** The identified miRNA-involved signaling pathways in both bioinformatic analysis ^1^.

Pathway	*p* Value	miRNA
P00057 Wnt signaling pathway	0.001861	miR-100-5p; miR-135a-5p; miR-137; miR-148a-3p; miR-192-5p; miR-194-5p; miR-195-5p; miR-203a-3p; miR-205-5p; miR-211-5p; miR-215-5p; miR-218-5p; miR-224-5p; miR-296-3p; miR-296-5p; miR-31-3p; miR-31-5p; miR-335-5p; miR-337-3p; miR-34b-3p; miR-34b-5p; miR-34c-5p; miR-376a-3p; miR-376a-5p; miR-376b-3p; miR-376c-3p; miR-380-5p; miR-485-5p; miR-9-5p; miR-935
WP673 ErbB signaling pathway	0.003884	miR-100-5p; miR-129-1-3p; miR-135a-5p; miR-192-5p; miR-194-5p; miR-199b-5p; miR-203a-3p; miR-205-5p; miR-20b-5p; miR-215-5p; miR-218-5p; miR-296-5p; miR-299-5p; miR-326; miR-335-5p; miR-34b-3p; miR-34b-5p; miR-34c-5p; miR-363-3p; miR-433-3p; miR-497-5p; miR-548d-3p; miR-654-3p; miR-99a-5p
P00034 Integrin signaling pathway	0.004683	miR-100-5p; miR-129-1-3p; miR-137; miR-148a-3p; miR-192-5p; miR-194-5p; miR-195-5p; miR-199b-5p; miR-205-5p; miR-215-5p; miR-218-5p; miR-224-5p; miR-296-3p; miR-31-3p; miR-31-5p; miR-326; miR-335-5p; miR-337-3p; miR-34b-5p; miR-34c-5p; miR-376a-3p; miR-433-3p; miR-497-5p; miR-9-3p; miR-9-5p; miR-935; miR-99a-5p
WP400 p38 MAPK Signaling Pathway	0.004683	miR-100-5p; miR-135a-5p; miR-137; miR-148a-3p; miR-192-5p; miR-194-5p; miR-195-5p; miR-203a-3p; miR-224-5p; miR-335-5p; miR-34b-3p; miR-34b-5p; miR-34c-5p; miR-433-3p; miR-99a-5p
hsa05211 Renal cell carcinoma	0.00488	miR-100-5p; miR-1262; miR-129-1-3p; miR-134-5p; miR-137; miR-192-5p; miR-194-5p; miR-195-5p; miR-199b-5p; miR-205-5p; miR-20b-5p; miR-215-5p; miR-218-5p; miR-224-5p; miR-296-3p; miR-31-5p; miR-335-5p; miR-337-3p; miR-34b-3p; miR-34b-5p; miR-34c-5p; miR-376a-3p; miR-377-3p; miR-433-3p; miR-489-3p; miR-497-5p; miR-9-5p; miR-935; miR-99a-5p
P00056 VEGF signaling pathway	0.007226	miR-100-5p; miR-134-5p; miR-137; miR-192-5p; miR-195-5p; miR-199b-5p; miR-205-5p; miR-20b-5p; miR-215-5p; miR-296-3p; miR-31-5p; miR-335-5p; miR-34b-3p; miR-376a-3p; miR-9-5p; miR-99a-5p
hsa04150 mTOR signaling pathway	0.007226	miR-100-5p; miR-134-5p; miR-192-5p; miR-195-5p; miR-199b-5p; miR-205-5p; miR-20b-5p; miR-215-5p; miR-218-5p; miR-296-3p; miR-31-3p; miR-335-5p; miR-34b-3p; miR-376a-3p; miR-9-5p; miR-99a-5p
hsa04350 TGF beta Signaling Pathway	0.007709	miR-100-5p; miR-153-3p; miR-192-5p; miR-194-5p; miR-195-5p; miR-203a-3p; miR-205-5p; miR-20b-5p; miR-215-5p; miR-218-5p; miR-224-5p; miR-299-5p; miR-31-3p; miR-335-5p; miR-337-3p; miR-409-3p; miR-433-3p; miR-9-5p; miR-935; miR-99a-5p
P00047 PDGF signaling pathway	0.024344	miR-100-5p; miR-135a-5p; miR-148a-3p; miR-192-5p; miR-194-5p; miR-195-5p; miR-20b-5p; miR-215-5p; miR-296-3p; miR-31-5p; miR-335-5p; miR-337-3p; miR-34b-3p; miR-34b-5p; miR-34c-5p; miR-376a-3p; miR-433-3p; miR-497-5p; miR-9-5p; miR-935; miR-99a-5p

^1^ The miRNAs in each pathway was identified through the miEAA website.

**Table 2:** The identified protein coding genes involved in biological process.

Gene Ontology Term	Count	*p* Value
GO:0007155~cell adhesion	110	1.74 × 10^−10^
GO:0022610~biological adhesion	110	2.18 × 10^−10^
GO:0030198~extracellular matrix organization	36	6.87 × 10^−9^
GO:0043062~extracellular structure organization	36	7.45 × 10^−9^
GO:0040007~growth	67	3.09 × 10^−8^
GO:0016477~cell migration	78	7.54 × 10^−8^
GO:0048589~developmental growth	48	8.20 × 10^−8^
GO:0000904~cell morphogenesis involved in differentiation	56	1.76 × 10^−7^
GO:2000145~regulation of cell motility	55	2.02 × 10^−7^
GO:0048870~cell motility	83	2.61 × 10^−7^
GO:0051674~localization of cell	83	2.61 × 10^−7^

**Table 3:** The association between miRNA expression and survival outcome.

miRNA	LogRank *p* Value	Type ^1^	Upregulated
hsa-miR-137	1.53 × 10^−2^	KIRC	Deceased
hsa-miR-153-3p	9.19 × 10^−4^	KIRC	Deceased
hsa-miR-153-3p	1.98 × 10^−3^	KIRP	Deceased
hsa-miR-224-5p	7.59 × 10^−5^	KIRP	Deceased
hsa-miR-224-5p	1.05 × 10^−2^	KIRC	Deceased
hsa-miR-296-3p	8.48 × 10^−5^	KIRC	Deceased
hsa-miR-296-5p	1.84 × 10^−4^	KIRC	Deceased
hsa-miR-335-5p	1.71 × 10^−3^	KIRC	Deceased
hsa-miR-34c-5p	3.75 × 10^−2^	KICH	Deceased
hsa-miR-34c-5p	1.98 × 10^−8^	KIRC	Deceased
hsa-miR-377-3p	4.07 × 10^−3^	KICH	Deceased
hsa-miR-377-3p	8.33 × 10^−6^	KIRC	Deceased

^1^ KICH, Kidney Chromophobe. KIRC, Kidney renal clear cell carcinoma. KIRP, Kidney renal papillary cell carcinoma.

**Table 4:** The association between miRNA expression and survival outcome.

microRNA (log2(Fold Change ^#^))	Target Genes (log2(Fold Change ^#^))	Experimental Validation	Reference
hsa-miR-137 (3.76)	CDC42 (−15.14)	Luciferase reporter assay	[36,37,38,39]
		qRT-PCR	
		Reporter assay	
		Western blot	
hsa-miR-137 (3.76)	LYPD6 (−3.90)	PAR-CLIP	[40,41,42,43,44]
hsa-miR-153-3p (2.65)	SNAI1 (−3.73)	Immunohistochemistry	[45,46,47,48]
		Luciferase reporter assay	
		qRT-PCR	
		Western blot	
hsa-miR-224-5p (6.88)	CDC42 (−15.14)	Luciferase reporter assay	[49,50,51]
		Microarray	
		qRT-PCR	
		Western blot	
hsa-mir-296-3p (−13.01)	CCAT1 (5.99) *	CLIP-Seq	[52]
hsa-mir-335-5p (6.16)	NEAT1 (−16.46) *	CLIP-Seq	[52]
hsa-miR-34c-5p (9.71)	NFE2L1 (−16.58)	PAR-CLIP	[43]
hsa-miR-34c-5p (9.71)	PPP1R11 (−4.25)	PAR-CLIP	[44]
hsa-miR-34c-5p (9.71)	NEAT1 (−16.46) *	CLIP-Seq	[52]
hsa-mir-377-3p (8.13)	SRSF1 (−17.83)	PAR-CLIP	[53]
hsa-mir-377-3p (8.13)	MTFR1L (−15.04)	PAR-CLIP	[41]
hsa-mir-377-3p (8.13)	NEAT1 (−16.46) *	CLIP-Seq	[52]

**#** fold change: ACHN vs. 786-O; * indicates non-coding RNA; HITS-CLIP (CLIP-Seq): High-throughput sequencing of RNA isolated by crosslinking immunoprecipitation; qRT-PCR: real-time reverse transcription-PCR; PAR-CLIP: photoactivatable ribonucleoside-enhanced crosslinking and immunoprecipitation.

**Table 5:** The association between miRNA expression and survival outcome.

GEO Accession Number	Gene Symbol	Fold Change	*p* Value
GSE22541	NEAT1	1.105	0.235
GSE22541	NFE2L1	0.684	<0.001
GSE22541	MTFR1L	0.850	0.115
GSE22541	SNAI1	0.886	0.194
GSE85258	NEAT1	0.941	0.224
GSE85258	NFE2L1	0.941	0.057
GSE85258	MTFR1L	0.806	0.030
GSE85258	SNAI1	1.032	0.758

## Supplementary Materials

The following are available online at https://www.mdpi.com/2075-4418/10/4/228/s1, Figure S1: RNA sequencing quality control report. The upper panel showed per base sequence quality of (**a**) miRNA from 786-O and ACHN, and (**b**) RNA from 786-O and ACHN; the lower panel showed the per sequence quality scores of (**c**) miRNA from 786-O and ACHN, and (**d**) RNA from 786-O and ACHN. The quality score from 15 to 99 bp fell into the green background, indicating good quality calls. The majority read > 35 indicated good sequencing quality; Table S1: The list of miRNA with significant changes (ACHN vs. 786-O); Table S2: The list of mRNA with significant changes (ACHN vs. 786-O); Table S3: The list of lncRNA with significant changes (ACHN vs. 786-O).

## References

[1] Siegel R.L., Miller K.D., Jemal A. (2019). Cancer statistics, 2019. CA Cancer J. Clin..

[2] Prasad S.R., Humphrey P.A., Catena J.R., Narra V.R., Srigley J.R., Cortez A.D., Dalrymple N.C., Chintapalli K.N. (2006). Common and uncommon histologic subtypes of renal cell carcinoma: Imaging spectrum with pathologic correlation. Radiographics.

[3] Athar U., Gentile T.C. (2008). Treatment options for metastatic renal cell carcinoma: A review. Can. J. Urol..

[4] Patard J.J., Leray E., Rioux-Leclercq N., Cindolo L., Ficarra V., Zisman A., De La Taille A., Tostain J., Artibani W., Abbou C.C. (2005). Prognostic value of histologic subtypes in renal cell carcinoma: A multicenter experience. J. Clin. Oncol..

[5] Moreno-Smith M., Lutgendorf S.K., Sood A.K. (2010). Impact of stress on cancer metastasis. Future Oncol..

[6] Ziello J.E., Jovin I.S., Huang Y. (2007). Hypoxia-Inducible Factor (HIF)-1 regulatory pathway and its potential for therapeutic intervention in malignancy and ischemia. Yale J. Biol. Med..

[7] Schodel J., Grampp S., Maher E.R., Moch H., Ratcliffe P.J., Russo P., Mole D.R. (2016). Hypoxia, Hypoxia-inducible Transcription Factors, and Renal Cancer. Eur. Urol..

[8] Rini B.I., Small E.J. (2005). Biology and clinical development of vascular endothelial growth factor-targeted therapy in renal cell carcinoma. J. Clin. Oncol..

[9] Altomare D.A., Testa J.R. (2005). Perturbations of the AKT signaling pathway in human cancer. Oncogene.

[10] Hudson C.C., Liu M., Chiang G.G., Otterness D.M., Loomis D.C., Kaper F., Giaccia A.J., Abraham R.T. (2002). Regulation of hypoxia-inducible factor 1alpha expression and function by the mammalian target of rapamycin. Mol. Cell Biol..

[11] Barata P.C., Rini B.I. (2017). Treatment of renal cell carcinoma: Current status and future directions. CA Cancer J. Clin..

[12] Mihaly Z., Sztupinszki Z., Surowiak P., Gyorffy B. (2012). A comprehensive overview of targeted therapy in metastatic renal cell carcinoma. Curr. Cancer Drug. Targets.

[13] Zerdes I., Tolia M., Tsoukalas N., Mitsis M., Kardamakis D., Pistevou-Gombaki K., Tsekeris P., Kyrgias G. (2019). Systemic therapy of metastatic renal cell carcinoma: Review of the current literature. Urologia.

[14] Tian T., Li X., Zhang J. (2019). mTOR Signaling in Cancer and mTOR Inhibitors in Solid Tumor Targeting Therapy. Int. J. Mol. Sci..

[15] Kim J., Yao F., Xiao Z., Sun Y., Ma L. (2018). MicroRNAs and metastasis: Small RNAs play big roles. Cancer Metastasis Rev..

[16] Mytsyk Y., Dosenko V., Skrzypczyk M.A., Borys Y., Diychuk Y., Kucher A., Kowalskyy V., Pasichnyk S., Mytsyk O., Manyuk L. (2018). Potential clinical applications of microRNAs as biomarkers for renal cell carcinoma. Cent. Eur. J. Urol..

[17] Braga E.A., Fridman M.V., Loginov V.I., Dmitriev A.A., Morozov S.G. (2019). Molecular Mechanisms in Clear Cell Renal Cell Carcinoma: Role of miRNAs and Hypermethylated miRNA Genes in Crucial Oncogenic Pathways and Processes. Front. Genet..

[18] Liu X., Hao Y., Yu W., Yang X., Luo X., Zhao J., Li J., Hu X., Li L. (2018). Long Non-Coding RNA Emergence During Renal Cell Carcinoma Tumorigenesis. Cell Physiol. Biochem..

[19] Li M., Wang Y., Cheng L., Niu W., Zhao G., Raju J.K., Huo J., Wu B., Yin B., Song Y. (2017). Long non-coding RNAs in renal cell carcinoma: A systematic review and clinical implications. Oncotarget.

[20] Bolger A.M., Lohse M., Usadel B. (2014). Trimmomatic: A flexible trimmer for Illumina sequence data. Bioinformatics.

[21] Kim D., Langmead B., Salzberg S.L. (2015). HISAT: A fast spliced aligner with low memory requirements. Nat. Methods.

[22] Friedlander M.R., Mackowiak S.D., Li N., Chen W., Rajewsky N. (2012). miRDeep2 accurately identifies known and hundreds of novel microRNA genes in seven animal clades. Nucleic. Acids Res..

[23] Backes C., Khaleeq Q.T., Meese E., Keller A. (2016). miEAA: microRNA enrichment analysis and annotation. Nucleic. Acids Res..

[24] Pathan M., Keerthikumar S., Ang C.S., Gangoda L., Quek C.Y., Williamson N.A., Mouradov D., Sieber O.M., Simpson R.J., Salim A. (2015). FunRich: An open access standalone functional enrichment and interaction network analysis tool. Proteomics.

[25] Huang da W., Sherman B.T., Lempicki R.A. (2009). Systematic and integrative analysis of large gene lists using DAVID bioinformatics resources. Nat. Protoc..

[26] Fan Y., Xia J. (2018). miRNet-Functional Analysis and Visual Exploration of miRNA-Target Interactions in a Network Context. Methods Mol. Biol..

[27] Chou C.H., Shrestha S., Yang C.D., Chang N.W., Lin Y.L., Liao K.W., Huang W.C., Sun T.H., Tu S.J., Lee W.H. (2018). miRTarBase update 2018: A resource for experimentally validated microRNA-target interactions. Nucleic. Acids Res..

[28] Vlachos I.S., Paraskevopoulou M.D., Karagkouni D., Georgakilas G., Vergoulis T., Kanellos I., Anastasopoulos I.L., Maniou S., Karathanou K., Kalfakakou D. (2015). DIANA-TarBase v7.0: Indexing more than half a million experimentally supported miRNA:mRNA interactions. Nucleic. Acids Res..

[29] Xiao F., Zuo Z., Cai G., Kang S., Gao X., Li T. (2009). miRecords: An integrated resource for microRNA-target interactions. Nucleic. Acids Res..

[30] Wong N.W., Chen Y., Chen S., Wang X. (2018). OncomiR: An online resource for exploring pan-cancer microRNA dysregulation. Bioinformatics.

[31] Tang Z., Kang B., Li C., Chen T., Zhang Z. (2019). GEPIA2: An enhanced web server for large-scale expression profiling and interactive analysis. Nucleic. Acids Res..

[32] Zheng G., Ma Y., Zou Y., Yin A., Li W., Dong D. (2018). HCMDB: The human cancer metastasis database. Nucleic. Acids Res..

[33] Xu Q., Krause M., Samoylenko A., Vainio S. (2016). Wnt Signaling in Renal Cell Carcinoma. Cancers.

[34] Liu X., Wang J., Sun G. (2015). Identification of key genes and pathways in renal cell carcinoma through expression profiling data. Kidney Blood Press Res..

[35] Feldkoren B., Hutchinson R., Rapoport Y., Mahajan A., Margulis V. (2017). Integrin signaling potentiates transforming growth factor-beta 1 (TGF-beta1) dependent down-regulation of E-Cadherin expression—Important implications for epithelial to mesenchymal transition (EMT) in renal cell carcinoma. Exp. Cell Res..

[36] Liu M., Lang N., Qiu M., Xu F., Li Q., Tang Q., Chen J., Chen X., Zhang S., Liu Z. (2011). miR-137 targets Cdc42 expression, induces cell cycle G1 arrest and inhibits invasion in colorectal cancer cells. Int. J. Cancer.

[37] Chen Q., Chen X., Zhang M., Fan Q., Luo S., Cao X. (2011). miR-137 is frequently down-regulated in gastric cancer and is a negative regulator of Cdc42. Dig. Dis. Sci..

[38] Zhu X., Li Y., Shen H., Li H., Long L., Hui L., Xu W. (2013). miR-137 inhibits the proliferation of lung cancer cells by targeting Cdc42 and Cdk6. FEBS Lett..

[39] Tamim S., Vo D.T., Uren P.J., Qiao M., Bindewald E., Kasprzak W.K., Shapiro B.A., Nakaya H.I., Burns S.C., Araujo P.R. (2014). Genomic analyses reveal broad impact of miR-137 on genes associated with malignant transformation and neuronal differentiation in glioblastoma cells. PLoS ONE.

[40] Memczak S., Jens M., Elefsinioti A., Torti F., Krueger J., Rybak A., Maier L., Mackowiak S.D., Gregersen L.H., Munschauer M. (2013). Circular RNAs are a large class of animal RNAs with regulatory potency. Nature.

[41] Lipchina I., Elkabetz Y., Hafner M., Sheridan R., Mihailovic A., Tuschl T., Sander C., Studer L., Betel D. (2011). Genome-wide identification of microRNA targets in human ES cells reveals a role for miR-302 in modulating BMP response. Genes Dev..

[42] Hafner M., Landthaler M., Burger L., Khorshid M., Hausser J., Berninger P., Rothballer A., Ascano M., Jungkamp A.C., Munschauer M. (2010). Transcriptome-wide identification of RNA-binding protein and microRNA target sites by PAR-CLIP. Cell.

[43] Krell J., Stebbing J., Carissimi C., Dabrowska A.F., de Giorgio A., Frampton A.E., Harding V., Fulci V., Macino G., Colombo T. (2016). TP53 regulates miRNA association with AGO2 to remodel the miRNA-mRNA interaction network. Genome Res..

[44] Hamilton M.P., Rajapakshe K.I., Bader D.A., Cerne J.Z., Smith E.A., Coarfa C., Hartig S.M., McGuire S.E. (2016). The Landscape of microRNA Targeting in Prostate Cancer Defined by AGO-PAR-CLIP. Neoplasia.

[45] Xu Q., Sun Q., Zhang J., Yu J., Chen W., Zhang Z. (2013). Downregulation of miR-153 contributes to epithelial-mesenchymal transition and tumor metastasis in human epithelial cancer. Carcinogenesis.

[46] Zhang Z., Sun J., Bai Z., Li H., He S., Chen R., Che X. (2015). MicroRNA-153 acts as a prognostic marker in gastric cancer and its role in cell migration and invasion. Onco Targets Ther..

[47] Wang Z., Liu C. (2015). MiR-153 regulates metastases of gastric cancer through Snail. Tumour. Biol..

[48] Zeng H.F., Yan S., Wu S.F. (2017). MicroRNA-153-3p suppress cell proliferation and invasion by targeting SNAI1 in melanoma. Biochem. Biophys. Res. Commun..

[49] Zhu S., Sachdeva M., Wu F., Lu Z., Mo Y.Y. (2010). Ubc9 promotes breast cell invasion and metastasis in a sumoylation-independent manner. Oncogene.

[50] Ke T.W., Hsu H.L., Wu Y.H., Chen W.T., Cheng Y.W., Cheng C.W. (2014). MicroRNA-224 suppresses colorectal cancer cell migration by targeting Cdc42. Dis. Markers.

[51] Zhang Y., Takahashi S., Tasaka A., Yoshima T., Ochi H., Chayama K. (2013). Involvement of microRNA-224 in cell proliferation, migration, invasion, and anti-apoptosis in hepatocellular carcinoma. J. Gastroenterol. Hepatol..

[52] Li J.H., Liu S., Zhou H., Qu L.H., Yang J.H. (2014). starBase v2.0: Decoding miRNA-ceRNA, miRNA-ncRNA and protein-RNA interaction networks from large-scale CLIP-Seq data. Nucleic. Acids Res..

[53] Whisnant A.W., Bogerd H.P., Flores O., Ho P., Powers J.G., Sharova N., Stevenson M., Chen C.H., Cullen B.R. (2013). In-depth analysis of the interaction of HIV-1 with cellular microRNA biogenesis and effector mechanisms. mBio.

[54] Wang M., Gao H., Qu H., Li J., Liu K., Han Z. (2018). MiR-137 suppresses tumor growth and metastasis in clear cell renal cell carcinoma. Pharmacol. Rep..

[55] Zhao S., Wang Y., Luo M., Cui W., Zhou X., Miao L. (2018). Long Noncoding RNA Small Nucleolar RNA Host Gene 1 (SNHG1) Promotes Renal Cell Carcinoma Progression and Metastasis by Negatively Regulating miR-137. Med. Sci. Monit..

[56] Wang K., Jin W., Jin P., Fei X., Wang X., Chen X. (2017). miR-211-5p Suppresses Metastatic Behavior by Targeting SNAI1 in Renal Cancer. Mol. Cancer Res..

[57] Jiang Y., Zhang H., Li W., Yan Y., Yao X., Gu W. (2020). FOXM1-Activated LINC01094 Promotes Clear Cell Renal Cell Carcinoma Development via MicroRNA 224-5p/CHSY1. Mol. Cell Biol..

[58] Wang K., Chen X., Zhan Y., Jiang W., Liu X., Wang X., Wu B. (2015). miR-335 inhibits the proliferation and invasion of clear cell renal cell carcinoma cells through direct suppression of BCL-W. Tumour. Biol..

[59] Xue D., Wang H., Chen Y., Shen D., Lu J., Wang M., Zebibula A., Xu L., Wu H., Li G. (2019). Circ-AKT3 inhibits clear cell renal cell carcinoma metastasis via altering miR-296-3p/E-cadherin signals. Mol. Cancer.

[60] Venneti S., Boateng L.A., Friedman J.R., Baldwin D.A., Tobias J.W., Judkins A.R., Mourelatos Z., Lal P. (2012). MiRNA-9 and MiRNA-200a distinguish hemangioblastomas from metastatic clear cell renal cell carcinomas in the CNS. Brain Pathol..

[61] Ning L., Li Z., Wei D., Chen H., Yang C. (2017). LncRNA, *NEAT1* is a prognosis biomarker and regulates cancer progression via epithelial-mesenchymal transition in clear cell renal cell carcinoma. Cancer Biomark..

[62] Liu Z., Chen Q., Hann S.S. (2019). The functions and oncogenic roles of *CCAT1* in human cancer. Biomed. Pharmacother..

[63] Chen S., Ma P., Li B., Zhu D., Chen X., Xiang Y., Wang T., Ren X., Liu C., Jin X. (2017). LncRNA CCAT1 inhibits cell apoptosis of renal cell carcinoma through up-regulation of Livin protein. Mol. Cell Biochem..

[64] Jiang B., Chen W., Qin H., Diao W., Li B., Cao W., Zhang Z., Qi W., Gao J., Chen M. (2019). TOX3 inhibits cancer cell migration and invasion via transcriptional regulation of SNAI1 and SNAI2 in clear cell renal cell carcinoma. Cancer Lett..

[65] Kim H.M., Han J.W., Chan J.Y. (2016). *Nuclear Factor Erythroid-2 Like 1 (NFE2L1)*: Structure, function and regulation. Gene.

[66] Chen S.C., Kuo P.L. (2016). Bone Metastasis from Renal Cell Carcinoma. Int. J. Mol. Sci..

[67] Bianchi M., Sun M., Jeldres C., Shariat S.F., Trinh Q.D., Briganti A., Tian Z., Schmitges J., Graefen M., Perrotte P. (2012). Distribution of metastatic sites in renal cell carcinoma: A population-based analysis. Ann. Oncol..

[68] Gudas L.J., Fu L., Minton D.R., Mongan N.P., Nanus D.M. (2014). The role of HIF1alpha in renal cell carcinoma tumorigenesis. J. Mol. Med..

[69] Chen S.C., Chen F.W., Hsu Y.L., Kuo P.L. (2017). Systematic Analysis of Transcriptomic Profile of Renal Cell Carcinoma under Long-Term Hypoxia Using Next-Generation Sequencing and Bioinformatics. Int. J. Mol. Sci..

[70] Lai X., Eberhardt M., Schmitz U., Vera J. (2019). Systems biology-based investigation of cooperating microRNAs as monotherapy or adjuvant therapy in cancer. Nucleic. Acids Res..

[71] Lai X., Gupta S.K., Schmitz U., Marquardt S., Knoll S., Spitschak A., Wolkenhauer O., Putzer B.M., Vera J. (2018). MiR-205-5p and miR-342-3p cooperate in the repression of the E2F1 transcription factor in the context of anticancer chemotherapy resistance. Theranostics.

[72] Byron S.A., Van Keuren-Jensen K.R., Engelthaler D.M., Carpten J.D., Craig D.W. (2016). Translating RNA sequencing into clinical diagnostics: Opportunities and challenges. Nat. Rev. Genet.

[73] Lai X., Wolkenhauer O., Vera J. (2016). Understanding microRNA-mediated gene regulatory networks through mathematical modelling. Nucleic. Acids Res..

