# Supplementary material for: Identification of the Potential Prognostic Markers from the miRNA-lncRNA-mRNA Interactions for Metastatic Renal Cancer via Next-Generation Sequencing and Bioinformatics
Source: Diagnostics (Basel). 2020 Apr 16;10(4):228. doi: 10.3390/diagnostics10040228 (PMC7235743; doi:10.3390/diagnostics10040228)
Supplement: Supplementary file 1 [file diagnostics-10-00228-s001.pdf]

Supplementary Materials

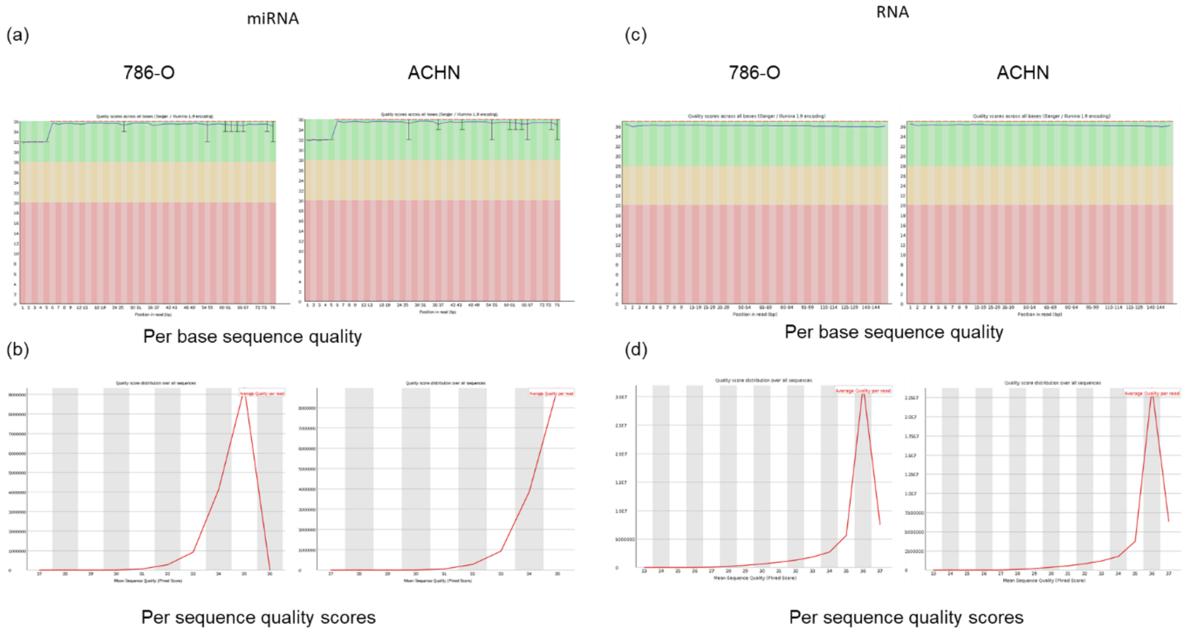

Figure S1. RNA sequencing quality control report. The upper panel showed per base sequence quality of (**a**) miRNA from 786-O and ACHN, and (**b**) RNA from 786-O and ACHN; the lower panel showed the per sequence quality scores of (**c**) miRNA from 786-O and ACHN, and (**d**) RNA from 786-O and ACHN. The quality score from 15 to 99 bp fell into the green background, indicating good quality calls. The majority read > 35 indicated good sequencing quality.

Table S1. The list of miRNA with significant changes (ACHN vs 786-O)

#miRNA	Fold Change	#miRNA	Fold Change	#miRNA	Fold Change
hsa-let-7a-2-3p	-490.00	hsa-miR-192-5p	21.37	hsa-miR-323a-3p	135.76
hsa-let-7c-3p	-10.30	hsa-miR-194-3p	31.18	hsa-miR-323b-3p	53.75
hsa-miR-100-3p	-26.39	hsa-miR-194-5p	21.87	hsa-miR-326	-16.46
hsa-miR-100-5p	-63.93	hsa-miR-195-5p	8.31	hsa-miR-329-3p	101.81
hsa-miR-105-5p	-288.00	hsa-miR-196a-3p	5.28	hsa-miR-329-5p	436
hsa-miR-1185-1-3p	32.08	hsa-miR-199b-5p	-9.75	hsa-miR-335-3p	73.67
hsa-miR-1185-2-3p	37.22	hsa-miR-203a-3p	222.25	hsa-miR-335-5p	71.58
hsa-miR-1185-5p	91.13	hsa-miR-205-5p	-5.12	hsa-miR-337-3p	1696
hsa-miR-1197	7.38	hsa-miR-20b-5p	5.28	hsa-miR-337-5p	12141
hsa-miR-125b-1-3p	-301.58	hsa-miR-211-5p	118	hsa-miR-34b-3p	1110.62
hsa-miR-125b-2-3p	5.91	hsa-miR-215-5p	18.88	hsa-miR-34b-5p	796.38
hsa-miR-1262	16.28	hsa-miR-218-5p	14.37	hsa-miR-34c-3p	10751
hsa-miR-1266-5p	6.62	hsa-miR-224-5p	118	hsa-miR-34c-5p	836.81
hsa-miR-127-3p	1723.39	hsa-miR-2277-3p	6.65	hsa-miR-3614-5p	141
hsa-miR-127-5p	4334.00	hsa-miR-2682-3p	6.63	hsa-miR-3617-5p	-140
hsa-miR-1271-5p	6.38	hsa-miR-296-3p	-8242.00	hsa-miR-363-3p	86.875
hsa-miR-129-1-3p	365.00	hsa-miR-296-5p	-3619.00	hsa-miR-365b-5p	-6.29
hsa-miR-129-5p	83.34	hsa-miR-299-3p	119.27	hsa-miR-3661	-11
hsa-miR-1291	5.29	hsa-miR-299-5p	125.68	hsa-miR-3679-5p	-9.77
hsa-miR-134-5p	114.22	hsa-miR-29b-1-5p	5.04	hsa-miR-3689a-5p	-171
hsa-miR-135a-5p	5.95	hsa-miR-3074-3p	-117	hsa-miR-3689b-5p	-171
hsa-miR-136-3p	1329.19	hsa-miR-3074-5p	-18.16	hsa-miR-3689e	-171
hsa-miR-136-5p	1145.25	hsa-miR-31-3p	-9550	hsa-miR-369-3p	131.57
hsa-miR-137	13.57	hsa-miR-31-5p	-23508.7	hsa-miR-369-5p	336.36
hsa-miR-148a-3p	8.61	hsa-miR-3115	-18.83	hsa-miR-370-3p	3227.00
hsa-miR-148a-5p	30.94	hsa-miR-3117-3p	1472	hsa-miR-370-5p	612
hsa-miR-153-3p	6.27	hsa-miR-3138	-5.78	hsa-miR-376a-2-5p	730
hsa-miR-154-3p	789	hsa-miR-3144-3p	-1751.00	hsa-miR-376a-3p	111.77
hsa-miR-154-5p	198.69	hsa-miR-3167	5.16	hsa-miR-376a-5p	95.63
hsa-miR-192-3p	20.09	hsa-miR-3189-3p	130	hsa-miR-376b-3p	125.43
hsa-miR-376b-5p	121.28	hsa-miR-4792	88.37	hsa-miR-548h-5p	36.28
hsa-miR-376c-3p	190.58	hsa-miR-4796-5p	23.5	hsa-miR-556-3p	7.07
hsa-miR-376c-5p	122.51	hsa-miR-4799-5p	130	hsa-miR-5583-3p	26.50
hsa-miR-377-3p	279.58	hsa-miR-4804-5p	23.50	hsa-miR-5584-5p	-148.00
hsa-miR-377-5p	306.00	hsa-miR-485-3p	178.64	hsa-miR-5701	-10.41

hsa-miR-379-3p	291.50	hsa-miR-485-5p	577	hsa-miR-574-3p	-5.60
hsa-miR-379-5p	100.23	hsa-miR-487a-3p	283	hsa-miR-584-3p	14.75
hsa-miR-380-3p	177	hsa-miR-487b-3p	161.59	hsa-miR-584-5p	5.79
hsa-miR-380-5p	648.00	hsa-miR-487b-5p	224	hsa-miR-585-3p	14.75
hsa-miR-381-3p	498.26	hsa-miR-489-3p	12.53	hsa-miR-616-5p	5.51
hsa-miR-381-5p	259.00	hsa-miR-491-3p	-202.00	hsa-miR-653-3p	10.26
hsa-miR-382-3p	191.38	hsa-miR-491-5p	-1564.00	hsa-miR-653-5p	13.38
hsa-miR-382-5p	169.78	hsa-miR-493-3p	21468.00	hsa-miR-654-3p	76.46
hsa-miR-409-3p	257.55	hsa-miR-493-5p	3769.75	hsa-miR-655-3p	1448
hsa-miR-409-5p	5181.00	hsa-miR-494-3p	86.53	hsa-miR-656-3p	412
hsa-miR-410-3p	11.06	hsa-miR-495-3p	153.81	hsa-miR-665	1613
hsa-miR-410-5p	294	hsa-miR-495-5p	39.75	hsa-miR-6844	-101.00
hsa-miR-411-3p	17.68	hsa-miR-496	342	hsa-miR-758-3p	203.13
hsa-miR-411-5p	117.27	hsa-miR-497-5p	5.33	hsa-miR-758-5p	61.87
hsa-miR-412-5p	60.38	hsa-miR-5010-3p	5.74	hsa-miR-767-5p	-265
hsa-miR-4286	-5.03	hsa-miR-539-3p	84.40	hsa-miR-770-5p	106.00
hsa-miR-431-3p	200.00	hsa-miR-539-5p	177	hsa-miR-874-3p	64.75
hsa-miR-431-5p	18371.00	hsa-miR-543	117.77	hsa-miR-874-5p	200
hsa-miR-432-5p	885.38	hsa-miR-544a	236.00	hsa-miR-889-3p	207.21
hsa-miR-433-3p	259.00	hsa-miR-544b	-265.00	hsa-miR-9-3p	-53.17
hsa-miR-4421	-202	hsa-miR-545-5p	-5.57	hsa-miR-9-5p	-37.67
hsa-miR-4636	-195	hsa-miR-548a-3p	-5.86	hsa-miR-92a-1-5p	6
hsa-miR-4662a-5p	-724.00	hsa-miR-548a-5p	-9.54	hsa-miR-935	-171.00
hsa-miR-4664-3p	-101	hsa-miR-548ak	173.75	hsa-miR-95-3p	5.59
hsa-miR-4766-5p	-8.41	hsa-miR-548aq-5p	-5.41	hsa-miR-99a-3p	-5.89
hsa-miR-4788	483	hsa-miR-548d-3p	-9.41	hsa-miR-99a-5p	-22.49

Table S2. The list of mRNA with significant changes (ACHN vs 786-O)

Gene symbol	Fold change	Gene symbol	Fold change	Gene symbol	Fold change
FN1	-26107300.0	TPI1	-218371.0	YBX3	-99767.1
EIF1AY	-5069170.0	KIAA1671	-210066.0	AC138811.2	-99293.1
TNFAIP2	-2353270.0	PSMD2	-201298.0	ZNF664	-99061.8
CPNE1	-2239300.0	UTP23	-193158.0	ANXA5	-98789.1
GOLM1	-1981890.0	UBC	-190611.0	DYNC1H1	-98406.2
FLNB	-1650300.0	PABPC1	-186530.0	NFE2L1	-98051.1
SH3BP2	-1519750.0	TBXAS1	-170578.0	EIF3E	-96634.1
PSMA5	-1075780.0	SNX14	-168960.0	ITSN1	-95498.4
DIDO1	-1036180.0	ALDOC	-160316.0	REPIN1	-94081.0
CARHSP1	-715470.0	TPGS2	-160022.0	CTSD	-93772.9
RBCK1	-534298.0	CD63	-152364.0	PAN2	-93639.5
SMAD9	-512966.5	SFPQ	-151901.0	DNMT1	-93112.3
MID1	-499277.0	ARHGDIA	-147733.0	LARGE1	-92977.5
TMBIM6	-486376.0	SYTL3	-146121.3	HSPG2	-92852.2
SEC31A	-486270.0	NCAPD2	-143943.0	SERF2	-92467.8
SAMD11	-474666.0	CNPY2	-143549.0	OGFOD1	-90846.7
WT1	-430349.0	CAP1	-142264.0	EPS8L2	-90235.7
ATXN2L	-423657.0	APP	-140822.0	SLC16A3	-88812.7
SMC4	-399850.0	RPS25	-139838.0	C5orf46	-84608.1
ECE1	-385611.0	NLGN4Y	-138263.0	LSM7	-84093.4
RPL30	-359880.0	SON	-126330.0	ATP5EP2	-83664.8
ZFY	-331733.0	COPA	-126023.0	TPR	-81369.8
NDRG1	-315488.0	HDLBP	-125851.0	FAM46A	-80411.8
UPK1B	-308784.0	IARS	-124384.0	TCTA	-78713.7
HSPA8	-297612.0	ZBTB38	-124231.0	TXNRD1	-76533.7
SLN	-272205.0	CEP250	-118985.0	BSG	-76123.9
SLITRK2	-260855.0	GART	-118101.0	WFDC3	-74585.6
IGFBP3	-256987.0	RTL3	-117121.0	CAST	-73595.1
ASPH	-254882.0	RACK1	-115778.0	SLC6A6	-73135.8
GNAS	-240939.0	PDIA3	-113162.0	TPRA1	-72579.3
IL17RA	-239973.0	KIAA0100	-110522.0	IQGAP1	-71913.7
SRSF1	-232735.0	SLC4A2	-109780.0	SLC3A2	-70235.3
TOP2A	-221999.0	PRKDC	-107340.0	RPL13	-70101.3
P4HB	-221332.0	EEF1E1	-101927.0	MASTL	-69317.9
PLS3	-219532.0	CSDE1	-100116.0	MCM4	-69142.6
VDAC1	-68917.8	HK1	-39315.8	HLA-A	-256.5

PLEKHG5	-68403.3	ANXA10	-39026.2	CDK15	-215.9
WWC1	-64792.8	SPANXB1	-37467.9	CAVIN3	-132.5
AC068580.4	-64273.6	WDR20	-37434.9	LPCAT2	-124.3
KIF2C	-60415.6	OSBPL9	-37194.5	ZNF736	-99.9
C1QBP	-59201.9	COMP	-37181.1	SEMA5A	-84.8
ANXA2	-59008.3	CDC42	-36111.1	ZNF718	-84.5
TMSB4Y	-58745.7	NARS	-35054.1	MAF	-78.2
HUWE1	-58090.5	NT5DC2	-34857.5	MYADM	-78.2
IL36B	-57813.6	CCDC194	-34635.6	INHBB	-67.3
RPL19	-56468.3	GMNC	-34345.0	ZNF426	-59.6
TXNDC9	-55232.9	PABPC5	-34182.1	VSTM4	-59.2
TMEM257	-54852.0	PDXK	-33836.5	HLA-B	-56.7
PITRM1	-53336.4	MTFR1L	-33813.4	TOX	-55.0
SLITRK4	-53287.9	IFNE	-33609.4	CLDN4	-48.4
PAX8	-52250.3	PTH2R	-33608.0	GATA2	-48.1
UNK	-51820.4	ERICH2	-32427.9	SLC7A7	-47.9
C11orf86	-51571.4	MATR3	-32412.9	SLC43A3	-47.0
SPP1	-51267.2	RAD17	-32301.9	SERPINE1	-46.4
LMNB1	-51022.9	MBL2	-32094.5	KLHL9	-46.2
AP2B1	-50922.8	KCNMA1	-30596.1	ZNF320	-41.9
ECD	-50017.0	SCUBE2	-30569.8	BST2	-40.8
MYO10	-50014.6	SULT1C2	-30316.3	ZNF518B	-37.4
CCL26	-49888.0	ELL3	-30196.3	ZNF600	-36.8
ALDOA	-49751.7	SULF1	-28871.7	ZNF788	-36.8
LRTOMT	-49701.6	PPIP5K1	-22695.2	G0S2	-36.3
DONSON	-49481.0	MAMLD1	-22397.2	ZNF93	-35.7
COL6A2	-47945.6	KCNJ3	-20700.2	ZNF844	-35.6
ENDOV	-47809.3	P2RY6	-18248.0	PLAC8	-35.5
LRFN5	-46045.8	ENG	-13048.5	ZNF28	-35.4
CRYZL1	-42937.1	SLCO2B1	-12860.0	ANPEP	-34.4
KIAA0895	-42736.3	KIF16B	-8075.6	NEDD9	-34.1
MUS81	-42529.6	RELB	-4456.0	GALNT3	-34.0
FLNA	-42281.0	UGT1A6	-939.7	MFNG	-32.4
TCP1	-41531.6	CXCL5	-622.2	FOXA2	-31.2
MCM7	-41387.1	PREX2	-460.7	ZNF440	-30.7
SAC3D1	-41130.5	ALPK3	-333.6	ATP8A1	-30.3
RPAIN	-40338.0	CDH6	-313.7	ZNF471	-29.3
SYT13	-28.5	TNFSF10	-14.5	CORO2B	10.6

ZNF441	-28.3	BMP2	-14.5	KCNIP3	10.7
FMN1	-27.2	ST3GAL1	-14.4	GRAMD1B	10.7
ECHDC3	-27.1	MMP13	-14.2	ABCC9	10.7
MAP7D2	-26.7	SIPA1L2	-14.2	FAM49A	10.8
ICAM1	-26.3	KIAA1024	-13.7	GATM	10.8
SLC16A2	-25.6	MMP2	-13.4	CDYL2	10.8
ATOH8	-25.6	SNAI1	-13.3	JPH2	10.9
TMEM173	-24.9	CA12	-13.3	HMGA2	11.1
DNAJC15	-23.9	COL6A3	-13.2	AC007906.2	11.1
MALL	-23.9	GPC4	-13.0	NKX6-1	11.1
HHIPL2	-22.8	PCDHB2	-12.9	NOVA1	11.2
MME	-22.2	ABCA1	-12.9	PRKG2	11.2
PTPRB	-21.5	SORBS1	-12.8	SPRED3	11.3
ACKR3	-20.1	ZNF365	-12.6	ITGB6	11.3
NUDT11	-19.9	RTN4RL2	-12.2	RNF182	11.4
KCNMB4	-19.6	BMF	-12.1	PADI2	11.4
ZNF737	-19.2	SPINT2	-11.9	FAM213A	11.4
PPP1R11	-19.0	KRT79	-11.8	PBX1	11.5
GFPT2	-18.7	CD99	-11.7	SIGLEC15	11.5
RNASET2	-18.5	ICOSLG	-11.5	SLC17A1	11.6
DGAT2	-18.2	OTUD1	-11.3	FGF12	11.7
TNFRSF11B	-17.8	IER3	-11.1	RRAGD	12.0
ADAM22	-17.2	APCDD1L	-10.9	GBX2	12.1
FGFR3	-17.1	RIPK4	-10.8	AIF1L	12.2
GPRIN3	-16.9	HECW2	-10.6	ZBED2	12.3
IL7R	-16.8	FOXL1	-10.6	FSD1	12.4
SNAI2	-16.7	ZNF83	-10.6	PRSS8	12.6
MMP16	-16.7	ITGB3	-10.5	SLC2A2	12.6
C9orf172	-16.6	CARD6	-10.2	CYP2J2	12.6
ZNF570	-16.3	CD70	-10.2	MYO1D	12.7
CFH	-16.0	DOCK4	10.0	RAB31	12.7
FLG	-15.7	NUP210	10.1	WTIP	12.8
BTBD11	-15.5	CCDC188	10.3	SLIT2	12.9
PER3	-15.4	CADM4	10.4	TAGLN3	13.1
WNT7B	-15.1	TTC22	10.4	KCNK2	13.2
BARX2	-15.1	MAPK13	10.4	ANTXR1	13.3
LYPD6	-15.0	MAP10	10.4	MPP2	13.3
BAIAP2L2	13.5	NPR2	17.2	NOV	27.3

TIAM1	13.6	NME4	17.3	FAM110C	27.6
ZNF474	13.7	SLCO4C1	18.1	PDE11A	27.9
NLRP3	13.8	RGS5	18.4	IL27RA	28.2
MATN3	14.0	SH3RF3	18.4	SLC24A2	29.1
CSF2	14.1	SIM2	18.7	NAT8L	29.4
PLCG2	14.1	FAM221A	18.8	CA11	29.5
PLPP2	14.2	PTK7	19.0	L1CAM	29.9
TEX15	14.2	CECR6	19.0	NFATC2	30.7
PARD6A	14.2	QRFPR	19.3	UGT2B7	30.9
ISYNA1	14.3	SLC25A33	19.4	SYT1	31.2
PPP1R3G	14.3	PNMA2	19.8	KCNQ3	31.5
ENPP4	14.3	TWIST2	19.8	PIK3AP1	32.0
KIF5A	14.6	SLC16A9	20.2	ACHE	34.0
ME3	14.7	RPS6KA6	20.6	CKB	35.0
HIF1A	14.8	KSR2	20.7	PPP2R2C	35.3
DYSF	14.8	GSPT2	21.0	IGDCC4	35.9
PLAU	14.9	PALD1	21.3	VANGL2	36.8
SYT16	15.0	CARD11	21.8	TESC	37.1
RIMS3	15.0	PAPPA2	21.8	GLDC	38.2
SALL2	15.0	FAM155B	22.0	HHEX	38.5
POU3F3	15.1	STK32B	22.3	LRRC3	39.1
EPHB1	15.3	KCTD15	22.3	ERICH5	39.5
C6orf132	15.4	BICDL1	22.4	CYP2S1	40.8
BFSP1	15.7	LOX	22.5	KCNH1	43.7
DISP2	15.7	RASSF6	23.6	ITGA4	44.7
TMEM98	16.0	CHST13	23.6	SV2A	45.1
SECTM1	16.0	ARMCX2	24.5	QPCT	45.1
CYBRD1	16.1	CCDC8	24.5	THY1	45.7
MDK	16.2	BATF3	24.8	NPNT	47.1
HOXB13	16.4	CXCL2	25.3	PAPPA	47.1
EPHB4	16.5	PKP2	25.4	ARMCX4	48.3
HSD17B14	16.6	EPHA10	26.1	HMGA1	50.1
METTL27	16.7	BTC	26.2	OGDHL	50.4
TDRP	16.7	CITED4	26.5	MARC1	54.0
GBGT1	16.9	FNDC10	26.9	ZNF853	54.9
TRIM32	17.0	ITPRIPL1	26.9	SEMA3E	56.9
RIMS2	17.2	SHISA9	27.3	KIAA1644	59.0
CCND2	59.5	DNAJC6	12357.9	PRRC2C	50958.9

SLC4A11	59.8	ANKRD36	24802.7	ENO3	54124.5
CXCL1	64.8	LMNA	30354.5	RPLP0	54412.5
FA2H	65.0	TCTEX1D1	30598.7	GCN1	54962.6
PHGDH	67.4	CTU2	30772.0	TPM1	56490.7
FAM83G	72.9	GCG	31113.6	ZNF385A	57679.8
EFNB3	73.2	TM9SF1	31530.8	SRP19	57982.0
SCIN	74.4	EFNA2	31575.4	KCNT2	58278.6
BCHE	85.2	USP34	31631.2	KARS	58790.0
MARCH4	87.6	FAM69A	31814.8	AKAP2	59499.6
WIPF3	88.5	PITX3	31905.8	EEFSEC	59786.9
KCNIP1	90.1	HPDL	32359.8	SMN1	60680.8
TBX18	92.5	NTS	32397.9	SAT2	61291.3
AFAP1L2	93.2	LARP1	32718.3	HSP90B1	61381.4
SLC34A2	107.3	SMG6	34665.4	ELP4	61382.8
COL4A4	111.6	IL31RA	34842.0	ARMC4	62403.6
VGF	140.8	ADRA2C	36027.8	PTGES3	62521.1
NPTX1	145.2	CHD4	37132.8	SP6	62656.8
SYT14	151.9	CYP1A2	37810.1	CCT3	62729.1
CPNE7	160.1	MAP3K12	38202.8	PRKAR1A	68026.6
FABP3	163.4	COL4A2	38278.6	ZC3H11A	68694.2
HS3ST3B1	183.6	KLRF1	38322.0	HOXD13	72741.9
LOXL4	193.1	HIST2H2AC	39500.5	PPME1	73924.9
NRK	195.7	MGAT5B	39668.2	IPO4	75408.0
PCDH20	214.9	ALDH3A1	40307.0	WLS	78139.7
LAMA1	229.2	EPB41L2	40667.2	DCBLD2	78285.7
ADRB2	268.3	PRRT2	40913.9	GRM5	79134.0
CAVIN2	334.6	DDX23	43403.9	ZNF362	79505.0
ADD2	335.4	NEURL4	45231.0	BSCL2	80123.7
KL	390.4	AP4M1	45680.9	LRRC23	80567.3
HS3ST3A1	404.3	CLPSL2	46103.5	NPC1	80607.3
CLDN16	1949.3	PTMA	46175.9	CCDC80	81511.7
WNT5A	7335.1	SNRPN	47257.8	PGAP2	82040.2
SPACA9	8191.8	TMEM25	48397.2	GAA	84277.1
RFX2	8521.5	SLC25A29	49123.3	NPM2	84514.1
RCBTB2	10093.2	ERBB3	49243.8	TUBA1B	85204.8
EXTL2	10323.2	AC106782.1	50599.1	TMEM138	85676.4
ZNF286B	11324.0	DYNAP	50798.2	PARP1	88524.9
CYB561A3	88541.2	NSD2	135550.0	CTNNA1	265704.0

PALM2-AKAP2	88644.4	RHNO1	137610.0	RPL6	272879.0
NPM1	89408.3	GOLGA4	139300.0	FOXL2NB	285257.0
NF1	89703.8	KRT8	143223.0	CHFR	298696.0
PIGL	90436.8	SLC38A1	143398.0	ALX1	313407.0
ADGRG1	91140.5	CALD1	143975.0	TJP2	320120.0
SMIM29	91712.6	SMARCC2	150883.0	CHEK1	320199.0
MOB1A	93634.9	NACA	155491.0	EIF4G1	340909.0
GOT2	97761.8	ENAH	163211.0	SEMA3C	341606.0
LDHA	98078.8	HNRNPUL1	163966.0	PPIA	341694.0
FAM159B	99532.3	MPDU1	164922.0	ZNF219	344990.0
CEP95	101456.0	EPB41L3	167402.5	MVP	346153.0
RPS3A	105752.0	FKBP11	170486.0	CDK16	348027.0
CDK5	106844.0	SCN2A	170546.0	FOXL2	398590.0
SCRN1	108265.0	FEZ2	176521.0	GAL3ST1	419652.0
ANKRD34B	108469.0	DLD	177416.0	MATN2	426513.0
POLE	108747.0	CORO1C	180867.0	UAP1	545610.0
YWHAZ	109982.0	SDC1	181535.0	CAV1	615854.0
CAD	115742.0	ANKRD11	189631.0	PHLDB2	764940.0
SRPK2	115981.0	CNOT1	193282.0	PSAP	784337.0
ACTB	117604.0	NUMA1	194011.0	DDX1	787163.0
FAT1	118337.0	GAL3ST3	202170.0	NXPH2	929075.0
HNRNPC	121174.0	CPSF4	206658.0	EIF4G2	1071790.0
SUN2	121632.0	CD44	212869.0	CAPG	1300790.0
KCTD10	124621.0	CREB3L2	221619.0	RPL14	1467890.0
TRABD2A	124675.0	MTHFD2	228076.0	NIN	1849350.0
TMEM123	126742.0	PML	234381.0	MT1M	2109760.0
VMP1	126782.0	SEPT7	246989.0	PADI3	2292310.0
LOXL2	128393.0	DDX5	256692.0	TM4SF4	2359260.0
USP44	132851.0	UBE2H	260097.0		
SOX6	134000.0	MOK	260194.0		

Table S3. The list of lncRNA with significant changes (ACHN vs 786-O)

Gene symbol	Fold change	Gene symbol	Fold change	Gene symbol	Fold change
AC067773.1	-3297440.0	AC092919.1	-45378.7	LINC01012	35234.2
TTTY15	-838070.0	AL445647.1	-43435.5	LINC00871	37800.2
LINC00704	-456892.0	AC100872.1	-42627.8	LINC02101	40353.2
AL713998.1	-399842.0	AC104257.1	-41917.4	LINC02310	40489.4
C22orf34	-351776.0	AC093635.1	-40606.6	AC068620.2	41370.8
LINC00278	-311985.0	AL121821.1	-39277.7	AC025219.1	41918.5
AC009159.2	-230009.0	AC084026.1	-38785.4	AF131215.7	43564.3
AL589669.1	-178085.0	AC016957.2	-35912.4	AC005034.4	48076.6
AC010889.1	-134975.0	AC103706.1	-33755.6	LINC01693	49469.7
AC009159.3	-126572.0	AC079148.1	-33642.1	LINC00472	49486.8
AL121936.1	-115886.0	C10orf126	-33094.7	AL021807.1	50779.9
AL589843.1	-110625.0	AL355432.1	-30968.1	LINC02453	51415.2
AL132655.2	-91968.6	AL645939.5	-30808.8	AC116345.1	52967.9
NEAT1	-90178.5	AL136162.1	-30580.5	BX322234.2	55440.6
AC034229.4	-84036.4	AC005014.2	-30171.7	AP003059.1	56440.3
AC018648.1	-82282.3	AC022034.2	-67.7	LINC02302	56461.2
AC080013.6	-81040.3	SNHG18	-52.3	AC079610.2	62630.3
LINCMD1	-80680.8	AL645608.3	-24.4	AC016924.1	66127.7
AC009271.1	-72670.9	ZNF667-AS1	-24.0	AL161630.1	66711.0
AC016717.2	-71518.5	LINC01508	-19.6	AC108865.1	70037.7
AP000894.4	-69623.6	AL645608.1	-16.0	MIR646HG	73107.7
AC069234.5	-59937.1	LINC00702	-15.3	AC112220.3	73119.5
MIR210HG	-59318.2	AC013451.2	10.7	CASC19	75183.3
TEX41	-58052.2	GAS6-AS2	14.2	AL133410.1	77017.1
AC097504.2	-56596.2	AC009237.14	15.4	U91319.1	86185.4
LINC01698	-55947.9	AL512274.1	17.3	AL133325.3	90369.6
AL023803.2	-53292.3	AC016705.2	20.8	LINC00452	97081.5
LINC01929	-51050.6	AC006033.2	32.0	SNHG25	97291.5
AC062004.1	-48757.2	CCAT1	63.6	AC009237.15	232356.0
AL132655.1	-48588.0	MEG3	161.0	AL138885.3	687848.0
LINC00441	-46222.4	AC017071.1	33206.7		

Table S4. The list of enriched biological processes (MiEAA, P value < 0.03)

Subcategory	miRNA count	p-value
WP1984 Integrated Breast Cancer Pathway	23	5.2574E-05
WP710 DNA damage response only ATM dependent	36	0.00081727
P00005 Angiogenesis	29	0.00118251
hsa04060 Cytokine cytokine receptor interaction	22	0.00118251
WP2034 Leptin signaling pathway	32	0.00150204
WP2064 Neural Crest Differentiation	21	0.00150204
hsa05416 Viral myocarditis	18	0.00172964
hsa04530 Tight junction	23	0.00181339
P00057 Wnt signaling pathway	30	0.00186094
WP304 Kit receptor signaling pathway	25	0.00186094
WP437 EGF EGFR Signaling Pathway	34	0.00186094
WP2256 Integrated Pancreatic Cancer Pathway	42	0.00201309
WP524 G13 Signaling Pathway	13	0.00201309
P00046 Oxidative stress response	14	0.00226826
WP1545 miRNAs involved in DDR	23	0.00226826
WP706 SIDS Susceptibility Pathways	16	0.0030573
hsa04510 Focal adhesion	39	0.00328513
P00049 Parkinson disease	13	0.00368536
WP1591 Heart Development	16	0.00368536
WP236 Adipogenesis	22	0.00368536
WP34 Ovarian Infertility Genes	13	0.00368536
WP45 G1 to S cell cycle control	29	0.00368536
hsa04115 p53 signaling pathway	29	0.00368536
P00036 Interleukin signaling pathway	24	0.00388377
WP138 Androgen receptor signaling pathway	31	0.00388377
WP179 Cell cycle	33	0.00388377
WP673 ErbB signaling pathway	24	0.00388377
hsa04310 Wnt signaling pathway	23	0.00388377
hsa04520 Adherens junction	30	0.00388377
hsa04110 Cell cycle	35	0.00422827
WP23 B Cell Receptor Signaling Pathway	25	0.00455941
P00034 Integrin signalling pathway	27	0.00468255
WP268 Notch Signaling Pathway	15	0.00468255
WP400 p38 MAPK Signaling Pathway	15	0.00468255
WP707 DNA damage response	28	0.00468255
WP712 Estrogen signaling pathway	15	0.00468255

hsa04330 Notch signaling pathway	15	0.00468255
hsa05200 Pathways in cancer	49	0.00468255
WP299 Nuclear receptors in lipid metabolism and toxicity	8	0.00487968
hsa04144 Endocytosis	29	0.00487968
hsa05211 Renal cell carcinoma	29	0.00487968
WP2037 Prolactin Signaling Pathway	31	0.00502689
hsa04722 Neurotrophin signaling pathway	31	0.00502689
P00006 Apoptosis signaling pathway	22	0.00532729
P00031 Inflammation mediated by chemokine and cytokine signaling pathway	25	0.00532729
WP306 Focal Adhesion	37	0.00532729
hsa04010 MAPK signaling pathway	32	0.00532729
hsa05120 Epithelial cell signaling in Helicobacter pylori infection	17	0.00610145
WP391 Mitochondrial Gene Expression	8	0.00689437
hsa04012 ErbB signaling pathway	27	0.00689437
hsa04621 NOD like receptor signaling pathway	10	0.00689437
hsa05219 Bladder cancer	27	0.00689437
P00056 VEGF signaling pathway	16	0.00722562
hsa04150 mTOR signaling pathway	16	0.00722562
WP382 MAPK signaling pathway	31	0.00725213
hsa04350 TGF beta signaling pathway	25	0.00725213
hsa05222 Small cell lung cancer	28	0.00733447
WP560 TGF beta Signaling Pathway2	20	0.00770942
hsa01100 Metabolic pathways	26	0.00814895
P00008 Axon guidance mediated by Slit Robo	7	0.00816998
WP286 IL 3 Signaling Pathway	21	0.00834297
WP411 mRNA processing	17	0.00834297
WP49 IL 2 Signaling pathway	21	0.00834297
WP75 Toll Like Receptor signaling	16	0.00834297
hsa04360 Axon guidance	21	0.00834297
hsa04620 Toll like receptor signaling pathway	16	0.00834297
hsa04062 Chemokine signaling pathway	26	0.00900714
P00012 Cadherin signaling pathway	15	0.00962553
hsa03040 Spliceosome	14	0.00993216
hsa04666 Fc gamma R mediated phagocytosis	17	0.0102403
P04393 Ras Pathway	17	0.0120176
hsa04630 Jak STAT signaling pathway	27	0.0120176

WP536 Calcium Regulation in the Cardiac Cell	12	0.0126025
hsa04514 Cell adhesion molecules CAMs	12	0.0126025
P00004 Alzheimer disease presenilin pathway	13	0.0126501
hsa05210 Colorectal cancer	29	0.0126501
WP615 Senescence and Autophagy	32	0.013412
hsa05212 Pancreatic cancer	34	0.0135354
P00054 Toll receptor signaling pathway	15	0.0141881
hsa04670 Leukocyte transendothelial migration	21	0.0141881
hsa05215 Prostate cancer	36	0.0141881
hsa04210 Apoptosis	20	0.0144206
WP2018 RANKL RANK Signaling Pathway	16	0.0153391
WP500 Glycogen Metabolism	6	0.0153391
hsa00450 Selenoamino acid metabolism	6	0.0153391
hsa03010 Ribosome	12	0.0153391
hsa03430 Mismatch repair	6	0.0153391
hsa04120 Ubiquitin mediated proteolysis	9	0.0153727
hsa05010 Alzheimers disease	15	0.016721
WP2035 FSH signaling pathway	12	0.0181076
hsa04020 Calcium signaling pathway	16	0.0181076
hsa04930 Type II diabetes mellitus	16	0.0181076
WP395 IL 4 signaling pathway	16	0.020975
hsa05218 Melanoma	31	0.020975
WP205 IL 7 signaling pathway	17	0.0210592
hsa03320 PPAR signaling pathway	8	0.0210681
P00029 Huntington disease	22	0.0218394
WP111 Electron Transport Chain	6	0.0218394
WP666 Hypothetical Network for Drug Addiction	9	0.0218394
hsa04912 GnRH signaling pathway	16	0.0218394
hsa05012 Parkinsons disease	10	0.0218394
hsa05020 Prion diseases	12	0.0218394
P00018 EGF receptor signaling pathway	20	0.0224635
P00052 TGF beta signaling pathway	19	0.0224635
WP1539 Angiogenesis	8	0.0224635
WP1544 MicroRNAs in cardiomyocyte hypertrophy	20	0.0224635
WP289 Myometrial Relaxation and Contraction Pathways	14	0.0224635
hsa00190 Oxidative phosphorylation	10	0.0224635
hsa00290 Valine leucine and isoleucine biosynthesis	5	0.0224635
hsa05221 Acute myeloid leukemia	19	0.0224635

hsa05340 Primary immunodeficiency	6	0.0224635
WP581 EPO Receptor Signaling	11	0.0233111
hsa04370 VEGF signaling pathway	16	0.0236472
P00059 p53 pathway	27	0.0237258
P00045 Notch signaling pathway	9	0.0243435
P00047 PDGF signaling pathway	21	0.0243435
P04398 p53 pathway feedback loops 2	24	0.0243435
WP185 Integrin mediated cell adhesion	21	0.0243435
P00021 FGF signaling pathway	17	0.025239
P00039 Metabotropic glutamate receptor group III pathway	5	0.0254464
P00048 PI3 kinase pathway	18	0.0254464
P04374 5HT2 type receptor mediated signaling pathway	8	0.0254464
P04391 Oxytocin receptor mediated signaling pathway	8	0.0254464
P04394 Thyrotropin releasing hormone receptor signaling pathway	8	0.0254464
P04396 Vitamin D metabolism and pathway	5	0.0254464
P05734 Synaptic vesicle trafficking	5	0.0254464
WP12 Osteoclast Signaling	5	0.0254464
WP244 Alpha 6 Beta 4 signaling pathway	13	0.0254464
WP357 Fatty Acid Biosynthesis	5	0.0254464
WP455 GPCRs Class A Rhodopsin like	8	0.0254464
WP477 Cytoplasmic Ribosomal Proteins	13	0.0254464
WP727 Monoamine Transport	5	0.0254464
hsa03030 DNA replication	5	0.0254464
hsa03060 Protein export	5	0.0254464
hsa03450 Non homologous end joining	5	0.0254464
hsa04260 Cardiac muscle contraction	8	0.0254464
hsa04512 ECM receptor interaction	13	0.0254464
hsa04672 Intestinal immune network for IgA production	5	0.0254464
hsa05223 Non small cell lung cancer	25	0.0254464
WP366 TGF beta Signaling Pathway1	29	0.0261272
hsa05214 Glioma	30	0.0261272
hsa05220 Chronic myeloid leukemia	34	0.0266608
P00038 JAK STAT signaling pathway	5	0.0270584
hsa04142 Lysosome	10	0.0276369
hsa05014 Amyotrophic lateral sclerosis ALS	16	0.0288746
hsa05130 Pathogenic Escherichia coli infection	17	0.0289515

Table S5. The list of enriched biological processes (DAVID, P value < 0.0001)

Gene ontology term	Count	P Value	Fold Enrichment	Bonferroni	Benjamini	FDR
GO:0007155~cell adhesion	110	1.74E-10	1.852211	1.08E-06	1.08E-06	3.39E-07
GO:0022610~biological adhesion	110	2.18E-10	1.845693	1.35E-06	6.74E-07	4.24E-07
GO:0030198~extracellular matrix organization	36	6.87E-09	3.083524	4.25E-05	1.42E-05	1.34E-05
GO:0043062~extracellular structure organization	36	7.45E-09	3.074319	4.61E-05	1.15E-05	1.45E-05
GO:0040007~growth	67	3.09E-08	2.056601	1.91E-04	3.82E-05	6.01E-05
GO:0016477~cell migration	78	7.54E-08	1.884665	4.66E-04	7.76E-05	1.47E-04
GO:0048589~developmental growth	48	8.20E-08	2.355396	5.07E-04	7.24E-05	1.60E-04
GO:0000904~cell morphogenesis involved in differentiation	56	1.76E-07	2.127572	0.001085	1.36E-04	3.42E-04
GO:2000145~regulation of cell motility	55	2.02E-07	2.137845	0.001248	1.39E-04	3.93E-04
GO:0048870~cell motility	83	2.61E-07	1.785327	0.001609	1.61E-04	5.07E-04
GO:0051674~localization of cell	83	2.61E-07	1.785327	0.001609	1.61E-04	5.07E-04
GO:0040011~locomotion	92	2.62E-07	1.720234	0.001615	1.47E-04	5.09E-04
GO:0010941~regulation of cell death	91	2.75E-07	1.725216	0.0017	1.42E-04	5.36E-04
GO:0043067~regulation of programmed cell death	86	4.34E-07	1.741196	0.00268	2.06E-04	8.45E-04
GO:0051270~regulation of cellular component movement	57	5.66E-07	2.033254	0.003492	2.50E-04	0.001101
GO:0042981~regulation of apoptotic process	85	5.68E-07	1.736929	0.003507	2.34E-04	0.001106
GO:0030334~regulation of cell migration	51	6.73E-07	2.129957	0.004152	2.60E-04	0.00131
GO:0040012~regulation of locomotion	55	7.61E-07	2.048768	0.004693	2.77E-04	0.001481
GO:0006928~movement of cell or subcellular component	101	1.30E-06	1.609712	0.008022	4.47E-04	0.002535
GO:0051216~cartilage development	22	1.72E-06	3.439243	0.010569	5.59E-04	0.003345

GO:0009611~response to wounding	48	1.83E-06	2.115864	0.011233	5.65E-04	0.003556
GO:0000902~cell morphogenesis	76	1.85E-06	1.753409	0.011382	5.45E-04	0.003604
GO:0030029~actin filament-based process	49	2.12E-06	2.086018	0.013004	5.95E-04	0.00412
GO:0007166~cell surface receptor signaling pathway	136	2.81E-06	1.457745	0.017242	7.56E-04	0.005475
GO:0009790~embryo development	62	3.29E-06	1.859236	0.020152	8.48E-04	0.006408
GO:0051130~positive regulation of cellular component organization	72	4.69E-06	1.741161	0.028597	0.00116	0.009133
GO:0010033~response to organic substance	141	4.86E-06	1.429399	0.029562	0.001153	0.009446
GO:0072358~cardiovascular system development	61	5.01E-06	1.84667	0.030486	0.001146	0.009746
GO:0072359~circulatory system development	61	5.01E-06	1.84667	0.030486	0.001146	0.009746
GO:0098609~cell-cell adhesion	71	5.24E-06	1.745005	0.031836	0.001155	0.010185
GO:0032989~cellular component morphogenesis	78	5.32E-06	1.689208	0.032334	0.001133	0.010347
GO:0008219~cell death	105	6.68E-06	1.53415	0.040448	0.001375	0.012997
GO:0051128~regulation of cellular component organization	120	7.14E-06	1.477826	0.043175	0.001423	0.013893
GO:0022603~regulation of anatomical structure morphogenesis	65	9.20E-06	1.769302	0.055272	0.001775	0.017898
GO:0061448~connective tissue development	24	9.22E-06	2.909313	0.055367	0.001725	0.017929
GO:0060560~developmental growth involved in morphogenesis	22	1.04E-05	3.070153	0.062377	0.001893	0.020274
GO:0030036~actin cytoskeleton organization	41	1.13E-05	2.121045	0.067577	0.001997	0.022024
GO:0060429~epithelium development	65	1.20E-05	1.754279	0.071407	0.002056	0.02332

GO:0048468~cell development	104	1.39E-05	1.511818	0.082453	0.002323	0.027086
GO:0080135~regulation of cellular response to stress	42	1.57E-05	2.068066	0.092475	0.00255	0.030542
GO:0048729~tissue morphogenesis	44	1.57E-05	2.023735	0.092559	0.002487	0.030571
GO:0048584~positive regulation of response to stimulus	106	1.87E-05	1.49383	0.1089	0.002878	0.03629
GO:0070887~cellular response to chemical stimulus	131	1.96E-05	1.415287	0.114327	0.002957	0.038212
GO:0007010~cytoskeleton organization	68	1.98E-05	1.70049	0.114968	0.002904	0.03844
GO:0001501~skeletal system development	37	2.22E-05	2.160215	0.128289	0.003188	0.043213
GO:0001525~angiogenesis	33	2.27E-05	2.285889	0.131112	0.003189	0.044233
GO:2001242~regulation of intrinsic apoptotic signaling pathway	18	2.32E-05	3.387819	0.133321	0.003175	0.045034
GO:0048646~anatomical structure formation involved in morphogenesis	68	2.36E-05	1.690148	0.135971	0.003172	0.045998
GO:0012501~programmed cell death	98	2.58E-05	1.513827	0.147577	0.003392	0.050253
GO:2001233~regulation of apoptotic signaling pathway	31	2.84E-05	2.333831	0.160822	0.003646	0.05518
GO:1902230~negative regulation of intrinsic apoptotic signaling pathway in response to DNA damage	8	2.93E-05	8.476518	0.165873	0.003695	0.05708
GO:0007369~gastrulation	19	3.31E-05	3.160213	0.185152	0.004087	0.064437
GO:2000026~regulation of multicellular organismal development	92	3.32E-05	1.529319	0.185698	0.00402	0.064648
GO:0006915~apoptotic process	93	3.50E-05	1.522935	0.19429	0.004146	0.067985
GO:0030155~regulation of cell adhesion	44	3.53E-05	1.957641	0.195911	0.004106	0.068619
GO:0040008~regulation of growth	44	4.84E-05	1.930618	0.258742	0.005529	0.094211

GO:0061138~morphogenesis of a branching epithelium	19	4.85E-05	3.070942	0.259063	0.005437	0.094347
GO:0001763~morphogenesis of a branching structure	20	5.44E-05	2.934179	0.285493	0.005985	0.10577
GO:0010769~regulation of cell morphogenesis involved in differentiation	28	6.11E-05	2.362923	0.314494	0.006603	0.118799
GO:0001568~blood vessel development	40	6.23E-05	1.986684	0.319721	0.00662	0.121206
GO:0045995~regulation of embryonic development	15	6.29E-05	3.636642	0.321939	0.006563	0.122233
GO:0016049~cell growth	33	6.43E-05	2.165303	0.327927	0.006601	0.125022
GO:0048638~regulation of developmental growth	26	6.46E-05	2.454833	0.329025	0.00652	0.125536
GO:0060548~negative regulation of cell death	55	6.49E-05	1.754129	0.33054	0.006451	0.126247
GO:0023056~positive regulation of signaling	84	6.76E-05	1.537487	0.341385	0.006607	0.131382
GO:0035295~tube development	40	7.24E-05	1.972983	0.360846	0.006969	0.140811
GO:0031589~cell-substrate adhesion	26	7.57E-05	2.430766	0.373595	0.00717	0.147145
GO:2000027~regulation of organ morphogenesis	23	7.69E-05	2.61107	0.378253	0.007174	0.149491
GO:0009967~positive regulation of signal transduction	78	7.73E-05	1.562635	0.379669	0.007102	0.150208
GO:0009887~organ morphogenesis	59	7.76E-05	1.701499	0.381099	0.007031	0.150933
GO:1902531~regulation of intracellular signal transduction	90	7.88E-05	1.502183	0.385659	0.007036	0.153257
GO:0042060~wound healing	38	8.13E-05	2.005744	0.395119	0.007156	0.158135
GO:0051094~positive regulation of developmental process	63	9.03E-05	1.655023	0.427654	0.007829	0.175511
GO:0022604~regulation of cell morphogenesis	39	9.88E-05	1.964299	0.45704	0.008446	0.192073
